# Novel and emerging anesthetic drugs for the treatments of major depression: a comprehensive review of efficacy, mechanism, and outlook

**DOI:** 10.3389/fpsyt.2025.1692751

**Published:** 2025-11-18

**Authors:** Congyi Li, Zhihui Wang, Xinman Ye, Jiahui Lv, Fangming Chen, Yining Zhang, Jianhua Liu, Xin Li, Jinnian Duan, Ying Wang, Bin Wang, Wei Tang, Jinghui Zhang, Yun Teng

**Affiliations:** 1 College of Basic Medicine, Dalian Medical University, Dalian, China; 2 Academy of Chinese Medical Science, Zhejiang Chinese Medical University, Hangzhou, Zhejiang, China; 3 School of Medicine, Shenzhen University, Shenzhen, China; 4 Department of Innovation and Entrepreneurship, Zhongshan College of Dalian Medical University, Dalian, Liaoning, China; 5 Department of Laboratory, The Second Affiliated Hospital of Dalian Medical University, Dalian, China; 6 Department of Radiation ,The Second Affiliated Hospital of Dalian Medical University, Dalian, China

**Keywords:** antidepressants, anesthetic agents, depression, ketamine, propofol, nitrous oxide, sevoflurane, isoflurane

## Abstract

Clinical and preclinical studies increasingly support the antidepressant potential of several anesthetic agents, including ketamine, propofol, nitrous oxide (N_2_O), sevoflurane, and isoflurane. Their therapeutic effects appear to arise from the regulation of multiple interconnected systems: modulation of glutamatergic and GABAergic signaling, interaction with monoaminergic neurotransmitters (5-HT, DA, NE), activation of neuropeptide-related pathways such as BDNF and VGF, regulation of the hypothalamic-pituitary-adrenal (HPA) axis, and suppression of inflammatory responses. These pathways overlap with core pathophysiological changes in depression and thus represent promising targets for intervention. Given the limited efficacy and delayed onset of traditional antidepressants, anesthetics with rapid antidepressant properties have emerged as attractive alternatives. However, their precise mechanisms of action, as well as questions regarding long-term safety and optimal clinical application, remain to be fully clarified. This review summarizes recent advances in both experimental and clinical research on the antidepressant effects of anesthetics, highlighting their underlying molecular and neural mechanisms, therapeutic potential, and current limitations. By integrating mechanistic insights with translational evidence, this article provides new perspectives and serves as a reference for future research aimed at developing safe and effective anesthetic-based therapies for treatment-resistant depression.

## 1 Introduction

Depression is one of the most prevalent and challenging mental disorders worldwide. According to the World Health Organization (WHO), the global prevalence of depression increased by about 26% during the COVID-19 pandemic (1–3). The WHO further projects that by 2030, depression will represent the leading cause of global disease of burden. Despite the availability of standard antidepressants, their efficacy remains limited, with only 20% to 30% of patients responding effectively and often with significant delays. Additionally, intolerable side effects lead many to discontinue treatment, highlighting the urgent need for novel, safe, rapid-acting antidepressants with fewer side effects.

Recent studies have shown that certain anesthetic agents exhibit antidepressant properties in addition to their conventional analgesic and sedative effects. However, the mechanisms underlying these effects remain unclear, limiting their translation into clinical practice. While most existing reviews have concentrated primarily on ketamine, this article takes a broader comparative approach by systematically examining propofol, nitrous oxide, sevoflurane, and isoflurane alongside ketamine. By bridging anesthesiology and psychiatry, it highlights both shared mechanisms and distinct pharmacological features, along with their potential clinical implications.

In this review, we first provide an overview of current knowledge on these anesthetic agents, followed by an exploration of their common molecular pathways and antidepressant mechanisms. We then assess their therapeutic potential, limitations, and future research directions. This structured approach aims to offer both mechanistic insights and translational perspectives to guide the development of anesthetic-based therapies for depression. In addition, this review is based on an extensive search of literature in PubMed and Web of Science databases prior to 2025, using terms such as “anesthetics,” “depression,” “ketamine,” “propofol,” “nitrous oxide,” “sevoflurane,” and “isoflurane. Both preclinical and clinical studies were included to highlight major molecular pathways and translational implications. Given the narrative nature of this work, no formal PRISMA protocol, inclusion/exclusion criteria, or risk-of-bias assessments were applied.

## 2 Ketamine: a prototype of rapid-acting antidepressants

Ketamine, one of the earliest developed anesthetics, has attracted considerable research interest since its antidepressant effects were first reported in 2000 (4). It has been shown to exert rapid and robust antidepressant effects, particularly in treatment-resistant depression (5–7.). In recent years, a growing body of clinical trials has demonstrated the clear advantages of ketamine over traditional antidepressants, especially following the FDA’s approval of esketamine nasal spray in 2019 as the first rapid-acting antidepressant (8, 9). However, concerns regarding side effects, including addiction and cognitive impairment, have also been raised (10). Therefore, it is of great importance to review the molecular mechanisms underlying ketamine’s antidepressant actions, and to compare its distinct pathways with those of other anesthetic agents such as nitrous oxide (N_2_O), propofol, sevoflurane, and isoflurane.

### 2.1 Modulation of glutamate and GABAergic systems

Glutamate, the most abundant excitatory neurotransmitter in the brain, plays a pivotal role in synaptic plasticity. Chronic stress can lead to excessive glutamatergic activity, impairing synaptic connectivity and subsequently disrupting the function of γ-aminobutyric acid (GABA), the principal inhibitory neurotransmitter. Abnormalities in either glutamate or GABA signaling-or more critically, an imbalance in glutamate/GABA-mediated excitatory-inhibitory (E/I) regulation across different brain regions-are considered central mechanisms in the pathophysiology of depression. Correcting this imbalance has therefore become a major focus of antidepressant development.

#### 2.1.1 NMDAR regulation and synaptic plasticity

Patients with depression exhibit impaired of glutamate-glutamine cycling in the anterior cingulate gyrus and prefrontal cortex (PFC), leading to excessive accumulation of glutamate and subsequent neurotoxicity, which contributes to the pathogenesis of depression (11). Glutamate-related receptors in humans include ionotropic receptors such as N-methyl-D-aspartate receptors (NMDARs) and AMPA receptors (AMPARs), as well as metabotropic G-protein–coupled receptors (mGluRs). While many studies have focused on glutamate receptors and their associated signaling pathways, the precise mechanisms underlying their role in depression remain to be fully elucidated. Recent progress has shed light particularly on NMDARs and mGluR2 in depression.

Excessive accumulation of extrasynaptic glutamate is thought to activate extrasynaptic NMDARs, triggering aberrant signaling that disrupts synaptic function and promotes neuronal loss. This process involves calcium influx through over-activated NMDARs, leading to excitotoxicity and neuronal death (12, 13), Such mechanisms are strongly implicated in depression (14, 15). Early studies also suggested a potential interaction between NMDARs and potassium channels (16). Thus, it is reasonable to hypothesize that ketamine may exert its fast-acting and sustained antidepressant effects by antagonizing the NMDAR modulation of potassium channel function.

Astrocytic glutamate transporter-1 (GLT-1), predominantly expressed in the hippocampus and cerebral cortex, plays a major role in regulating extrasynaptic glutamate levels (17, 18). By enhancing GLT-1 expression via the NMDAR-BDNF-TrkB pathway, ketamine promotes glutamate uptake by astrocytes, reduces extrasynaptic glutamate concentration, prevents neuronal overactivation, and thereby exerts antidepressant effects (19).

Postsynaptic density protein 95 (PSD-95), a key structural component of excitatory synapses, regulates receptor expression and synaptic plasticity (20). Clinical evidence suggests a close association between reduced PSD-95 levels and depression. NMDAR-dependent long-term depression (LTD) is accompanied by autophagy-mediated loss of PSD-95, which alters synaptic plasticity (21). Multiple clinical studies have shown a close association between decreased PSD-95 protein and the development of depression. PSD-95 protein is involved in the regulation of multiple neurotransmitter receptors and ion channels, and Early growth response 1 (Egr-1) is a negative regulator of PSD95 protein. Ketamine has been shown to increase PSD-95 expression by downregulating Egr-1, a negative regulator of PSD-95, through blockade of NR2B-containing NMDARs (22). This restoration of PSD-95 enhances synaptic plasticity and may underlie ketamine’s rapid antidepressant effects.

Glutamate binding to NMDAR, AMPAR initiates postsynaptic membrane depolarization, causing inward calcium ions flow, subsequent CaMKII activation, and AMPAR phosphorylation (21). The activated CaMKII affects synaptic plasticity, and phosphorylates Neuroligin 1 to increase its surface expression, promoting the new synapse formation (23). Calcium influx also suppresses microglial activation and NLRP3 inflammasome activity, reducing neuroinflammation and contributing to antidepressant effects (24). By antagonizing NMDAR-mediated calcium entry, ketamine decreases neuronal hyperexcitability, inhibits inflammatory responses and oxidative stress, and slows the progression of depression.

Additionally, ketamine has been reported to enhance AMPAR expression by inhibiting NLRP3 activation, further reinforcing its antidepressant effects (25, 26). Collectively, these findings suggest that ketamine exerts antidepressant actions through multiple NMDAR- and AMPAR-related mechanisms, ultimately promoting synaptic resilience and reducing neuroinflammation.

#### 2.1.2 AMPAR activation and rapid antidepressant response

AMPA receptors (AMPARs), ionotropic glutamate receptors composed of four subunits (GluA1–GluA4), are located on the postsynaptic membrane of excitatory glutamatergic synapses and are closely linked to synaptic plasticity. Ketamine indirectly activates AMPARs by antagonizing NMDARs and thereby increasing extrasynaptic glutamate concentrations. In addition, ketamine can directly activate AMPARs, rapidly modulating neuronal excitability and producing antidepressant effects. For example, ketamine activates Rac1, which promotes AMPAR recruitment to the postsynaptic membrane via the BDNF pathway, enhancing excitatory postsynaptic potentials and alleviating depressive behavior (27). These findings support the hypothesis that AMPAR activation triggers rapid downstream BDNF signaling responses, resulting in ketamine’s rapid antidepressant effect.

The metabotropic glutamate receptor 2 (mGluR2), located on presynaptic terminals, acts as an inhibitory receptor that regulates glutamate release (28). In both the chronic unpredictable mild stress (CUMS) and chronic restraint stress (CRS) models, mGluR2 has been implicated in antidepressant mechanisms (29, 30), While most clinical data suggest decreased glutamate levels in the brains of depressed patients, some studies show that upregulation of mGluR2, leading to reduced glutamate release, also exerts antidepressant effects—contradicting earlier findings (31). For example, Elhussiny et al. reported that ketamine upregulated mGluR2 expression and exerted antidepressant effects (32). This may reflect stress-induced glutamate over-release, which contributes to depression-related neuropathology; thus, increasing mGluR2 may help restore homeostasis. These findings suggest that early intervention with ketamine, even prior to depressive onset, may help prevent disease development.

Antagonists of mGluR2 also exhibit antidepressant properties through mechanisms overlapping with those of ketamine, including increased glutamate release, enhanced excitatory synaptic activity (33), AMPAR activation (34), mTOR pathway activation (35), and promotion of synapse-relaed protein synthesis (36). Furthermore, studies by Zanos et al. revealed that hydroxynorketamine (HNK), a metabolite of ketamine, exerts antidepressant effects dependent on mGluR2 signaling (28), This suggests that ketamine’s antidepressant efficacy may involve not only direct receptor interactions but also its metabolites acting on mGluR2. Nonetheless, the precise molecular mechanisms underlying ketamine–mGluR2 interactions remain to be clarified.

#### 2.1.3 Restoration of GABAergic transmission and parvalbumin interneurons

GABA, the brain’s main inhibitory neurotransmitter, is synthesized from glutamate by GAD. Reduced activity of GABAergic neurons leads to glutamate accumulation in the synaptic cleft, causing excitotoxicity due to impaired conversion. Clinical evidence supports this mechanism: neuroimaging studies reveal decreased GABA levels in the PFC, occipital cortex, and cingulate gyrus of depressed patients (37, 38), while postmortem studies show fewer GABAergic neurons in the PFC (39). Findings indicate that reduced GABA levels and diminished GABA neuron populations are closely linked to depression. Ketamine may exert antidepressant effects by restoring the glutamate-GABA-glutamine cycle between cortical neurons and astrocytes via NMDAR modulation, although the upstream mechanisms remain to be elucidated (19).

The medial prefrontal cortex (mPFC) is a vital brain region in which ketamine exerts its tachyphylactic antidepressant effects (32), especially in layer V pyramidal cells (14). Chronic unpredictable stress (CUS) reduces synaptic proteins, dendritic spines, and excitatory postsynaptic current strength in these neurons. Ketamine rapidly reverses these structural and functional deficits by promoting spine and synapse formation in an mTOR-dependent manner. In the CUMS model, presynaptic GABA synthesis, release, and uptake in the mPFC are also reduced. Ketamine dose-dependently increases GABA release from the mPFC, elevates GABA levels in the anterior cingulate gyrus, and alleviates depressive behavior (37). These findings suggest that ketamine restores excitatory–inhibitory balance, potentially via perineuronal nets (PNNs) in the prelimbic cortex, which are essential for GABAergic neuron function and synaptic plasticity (40, 41).

Parvalbumin (PV) has gained extensive research interest for its role in depression as a subtype of GABAergic neurons in recent years. Experimental evidence has pinpointed the Glutamate receptor N-methyl-D-aspartate 2A (GLUN2A) on parvalbumin mediates the immediate effects of low doses of ketamine (42), while the GLUN2B-NMDAR on GABAergic interneurons stand as the focal point for the fast-acting antidepressant effects of ketamine (43). Interestingly, a recent study reported that GluN2A on excitatory neurons may serve as the main target for ketamine, providing rapid antidepressant effects without psychiatric side effects (44). PNNs, which enwrap PV neurons, provide structural and functional support; chronic mild stress (CMS) reduces PNN density, increasing vulnerability to stress (45). Experimental removal of PNNs or knockdown of Neurocan, a core PNN component, increases stress susceptibility in rodents; while Neurocan overexpression confers resilience (46). Ketamine enhances Neurocan expression within PNNs, restores PV^+^ neuron function, and alleviates depressive behaviors, particularly in adolescent models (46, 47).

Stress paradigms appear to differentially affect GABA transmission: acute stress enhances hippocampal GABAergic synaptic activity, whereas chronic stress reduces it (48). Therefore, further studies are needed to delineate how ketamine’s antidepressant mechanisms vary under different stress conditions.

### 2.2 Neurotrophic and anti-inflammatory pathways

Brain-derived neurotrophic factor (BDNF) plays a central role in the pathophysiology of depression and in antidepressant responses. By activating TrkB receptors, BDNF promotes neuronal survival, synaptic plasticity, and neural repair through multiple intracellular signaling cascades (49). Increasing evidence indicates that ketamine enhances BDNF expression, activates the ERK-CREB pathway, and upregulates glucose transporter 3 (GLUT3), thereby improving astrocytic glucose uptake. These processes enhance neuronal metabolism and are thought to underlie ketamine’s antidepressant actions. It is noteworthy that most of the glucose entering the brain undergoes metabolism from glutamate, a precursor of GABA as well. Increased glucose utilization is beneficial to maintain the balance between glutamate and GABA, exerting an antidepressant role (50). This linkage thereby establishes a connection between the BDNF-TrkB-ERK-mTOR1-CREB signaling pathway and the glutamatergic and GABAergic doctrines of depression. Contrary to the above findings, ERK-ERK1/2 signaling pathway is over-activated as an inflammatory signaling pathway in patients with depression, whereby ketamine exerts neuroprotective and antidepressant effects by inhibiting this process (51). These inconsistencies may reflect differences in treatment duration or experimental conditions.

The BDNF-TrkB pathway also regulates classical monoaminergic neurotransmitters, including serotonin, dopamine, and norepinephrine, and interacts with PI3K/Akt and mTOR signaling to coordinate cell growth and metabolism. Moreover, BDNF has strong anti-inflammatory effects (52). Activated inflammatory factors lead to a decrease in BDNF (53), whereas ketamine elevates BDNF and simultaneously suppresses inflammation, thereby improving depressive behaviors (54). This anti-inflammatory action may itself be mediated through BDNF-TrkB signaling (55).

Ketamine’s effects entail an increase in the release not only of BDNF but also of transforming growth factor-beta 1 (TGF-β) (56). TGF-β1 is an anti-inflammatory factor that plays a neuroprotective role in many neurological disorders (57). Deficiency in TGF-β1 can lead to depression (58). Clinical studies have shown reduced plasma TGF-β1 in patients with major depressive disorder (MDD), which correlates with depression severity (58, 59). Reduced TGF-β1 contribute to drug resistance, while higher TGF-β1 favors antidepressant medication (32). Impaired TGF-β1 signaling has also been shown to impair synapse formation and synaptic plasticity in mice (60), as well as induce depressive behavior (61) in animal models. It has been demonstrated that (R) ketamine rapidly ameliorates chronic social defeat stress (CSDS)-induced reduction of spine density in the mPFC and hippocampus, eliciting an antidepressant effect (62). This effect may be due to the induction of synapse-associated protein synthesis through activation of the ERK-NRBP1-CREB-BDNF pathway in microglial cells and enhancement of synaptic plasticity (63). Conversely, TGF-β increases CREB protein phosphorylation (64), thereby not only enhancing synaptic excitability in the short term, but also exerting a long-lasting effect on synaptic plasticity. Another experiment demonstrated that TGF-β1 receptor on microglia and its downstream signaling pathway mediate the antidepressant effects of R ketamine in CSDS mice (65).

Hyperactivation of the hypothalamic-pituitary-adrenal (HPA) axis and inflammation are strongly associated with depression (66). Hyperactivation of the HPA axis leads to increase secretion of cortisol and corticosterone from the adrenal glands (67). Of these, glucocorticoids play a key role in the development of depression. Although glucocorticoids are generally recognized for their anti-inflammatory role, studies have shown that they can also play a pro-inflammatory role under both acute and chronic stress. Overactivation of the HPA axis increases glucocorticoid levels, which in turn promote the expression of pro-inflammatory factors, mainly NLRP. Additionally, elevated glucocorticoid can block the negative feedback inhibition of the HPA axis through the genome effect of glucocorticoid receptors (GR), thus creating an over-activated HPA axis (68). Consequently, a vicious circle of HPA axis overactivation is formed. Pro-inflammatory cytokines activate microglia and promote differentiation of microglia towards the M1 phenotype, whereas M1-type microglia secrete cytokines to further amplify inflammation and drive depression (69, 70). The study by Liu Y et al. used a drug delivery system that targeted microglia and found that inhibition of microglial MPA axis was not possible (70). LeGates et al. found that inhibition of microglia M1-type polarization was effective in relieving inflammation-related, consistent with the above view (71). Meanwhile, increased corticosterone are integral to the progression of depression (72). Ketamine reduces peripheral corticosterone concentration, normalizes corticosterone receptor expression, and facilitates synapse formation, exerting an antidepressant effect (73).

The NLRP inflammasome is a three-part multiprotein complex whose activation is associated with microglia-mediated neuroinflammation and partial neuronal degeneration.The NLRP1 and NLRP3 inflammasomes are mainly expressed in microglia of the brain (74, 75). NLRP1-driven inflammatory responses were shown to be involved in chronic stress-induced depressive behaviors, which may be related to the CXCL1-CXCR2-BDNF signaling pathway (75), and ketamine can exert rapid antidepressant effects mediated through the BDNF pathway (76). In addition, numerous studies have shown that CUMS leads to a significant increase in NLRP3 in mice (32, 77), which promotes neurotoxic glial activation and depressive phenotypes (78). Ketamine exerts a rapid antidepressant effect by inhibiting NLRP3 inflammatory vesicle activation (79, 80). This highlights a possible interaction between ketamine, NLRP inflammasomes, and BDNF pathways. Recent evidence also implicates the SIK1-CRTC1 signaling pathway in PVN neurons mediates CSDS- and CUMS-induced depressive behaviors (79, 81), and that ketamine enhances CRTC1 expression and induces enhanced excitatory synaptic transmission at Schaffer side branch CA1 synapses, exerts rapid antidepressant effects, and ameliorates depressive behavior in CRTC knockout mice (82). These findings further support a link between ketamine’s rapid antidepressant effects, the HPA axis, and neuroplasticity.

Both over-activated HPA axis and inflammation are involved in the development of depression (83) and they interact with each other. Namely, the abnormal HPA axis under chronic stress causes activation of the immune system, and chronic stress of the immune system triggers low-grade inflammation. Elevated cytokines released during inflammation, such as IGF and other inflammatory markers, will affect neurotransmitter and neurotrophic regulation, thereby reducing neurogenesis and participating in the development of depression (84).

### 2.3 Other neurotrophic factors (BICC1, VGF, IGF family)

BICC1 is considered a downstream signal of BDNF-TrkB-mTOR pathway and plays a role in regulating GluA1 expression (85). Necropsy revealed that BICC1 mRNA expression is upregulated in the dorsolateral prefrontal cortex and dentate gyrus of MDD patients. Similarly, increased BICC1 expression has been observed in prefrontal cortex and hippocampal regions of CUMS mice. Notably, BICC1 gene knockdown has effective in preventing the development of depressive behaviors (86). Genetic studies further support this link, with two single nucleotide polymorphisms (SNPs) in the BICC1 gene associated with depression (87). Ketamine treatment rapidly decreases BICC1 expression, correlating with the reversal of depressive behaviors in animal models (88).

VGF is a secreted protein and neuropeptide precursor regulated by BDNF and involved in synaptic plasticity (89). Research has linked VGF and its derivatives to depression mechanisms (90). In the CSDS mice, VGF produces antidepressant effects and promotes cell proliferation in hippocampal dentate gyrus (91), which itself contributes to ameliorating depressive behaviors. VGF mediates ketamine’s rapid antidepressant effects through the TrkB-mTOR-BICC1 signaling pathway, specifically by regulating GluA1 phosphorylation (92). Ketamine’s prevention of CRS-induced 4E-BP1 phosphorylation, PSD-95 and GluA1 immunocontent in the prefrontal cortex, reinforcing its characteristic of a prophylactic agent to manage individuals at-risk to develop MDD and anxiety (93). This process may be mediated by VGF-enhanced synaptic transmission via (94), promotion of dendritic maturation (95), and induction of synaptogenesis (96).

The VGF-derived peptide TLQP-62 also exhibits antidepressant effects when administered into the hippocampus (97). Its actions appear to involve activation of the BDNF-TrkB-CREB pathway (94) and induction of neurogenesis via NMDAR and mGluR5 signaling (98). TLQP-62 transiently increases tissue plasminogen activator (tPA) levels (98), promoting the conversion of proBDNF to mature BDNF (mBDNF) in the hippocampus, which further contributes to antidepressant outcomes (99). In the ventromedial prefrontal cortex (vmPFC), VGF modulates susceptibility to CRS and ketamine’s antidepressant efficacy, through mechanisms involving BDNF expression and calcium signaling (100). Collectively, these findings suggest that VGF and its peptides act as important mediators of ketamine’s rapid antidepressant effects by supporting neurogenesis and synaptic plasticity.

Insulin-like growth factor-1 (IGF-1) is another neurotrophic factor essential for synaptic transmission and plasticity in the central nervous system (101). Reduced IGF-1 levels could be a potential biomarker of depression in animal models (102). Conversely, increasing IGF-1 levels in the brain exerts antidepressant effects (103). In lipopolysaccharide (LPS)–induced depression models, ketamine’s antidepressant effects were shown to depend on IGF-1 release in the medial prefrontal cortex (104).

Similarly, IGF-2 expression is downregulated in the hippocampus of mice exposed to CUS (105) and CRS (106). In contrast, ketamine at antidepressant doses increased IGF-2 and p11 expression, promoting neuronal progenitor cell proliferation and yielding antidepressant effects (106, 107). Thus, ketamine may exert rapid antidepressant effects by enhancing IGF signaling, supporting synaptic plasticity, and counteracting neuroinflammation.

### 2.4 Autophagy, mitophagy, and cellular homeostasis

Autophagy, a conserved intracellular degradation pathway, plays a key role in maintaining cellular homeostasis and is generally considered cytoprotective. Chronic restraint stress suppresses hippocampal neurogenesis in mice by inducing autophagic cell death (ACD) in neural stem cells (NSCs) (108). Similarly, chronic stress has been shown to inhibit autophagy in rats (109) and promote iron-dependent neuronal death in the hippocampus (110).

Synaptic plasticity is also linked to autophagy. NMDAR-dependent long-term depression (LTD) promotes autophagy-mediated removal of phosphorylated PSD-95 (at T19), which increases AMPAR surface mobility and enhances short-term plasticity (21). Conversely, inhibition of autophagy during LTD reduces AMPAR endocytosis, thereby preserving the AMPAR ratio in the postsynaptic membrane (111). These findings suggest that autophagy may improve synaptic plasticity by modulating postsynaptic AMPAR through different autophagic mechanisms levels during LTD (112). On the other hand, autophagy has also been reported to promote AMPAR degradation, further highlighting its context-dependent effects on synaptic regulation (55). Autophagy also interacts with inflammatory pathways relevant to depression. The NLRP3 inflammasome, a critical mediator linking stress to inflammation, is upregulated in patients with depression and promotes release of pro-inflammatory cytokines. Autophagy inhibits excessive NLRP3 activation, thereby reducing cytokine release, attenuating systemic inflammation, and improving depressive symptoms (113).

Ketamine appears to influence autophagy in ways that improve both synaptic plasticity and inflammation. Studies show that ketamine stimulates autophagy by increasing levels of LC3II and ATG5, while reducing ATG4 and p62/SQSTM1, collectively promoting autophagic activity (109). These effects are associated with improved hippocampal neuroplasticity in stress-exposed rats. Ketamine also protects mitochondrial function by preventing TNF-α–induced degradation of NIX (NIP3-like protein X), thereby enhancing mitophagy and alleviating synaptic deficits (114). Furthermore, ketamine reduces NLRP3-driven inflammation by enhancing hippocampal autophagy, decreasing oxidative stress, and providing neuroprotection (25, 78, 115). It also reverses LPS-induced microglial autophagy blockade by upregulating the HMGB1–RAGE axis (116) and protects against ferroptosis-related cell death in the hippocampus (109).

**Figure 1:**
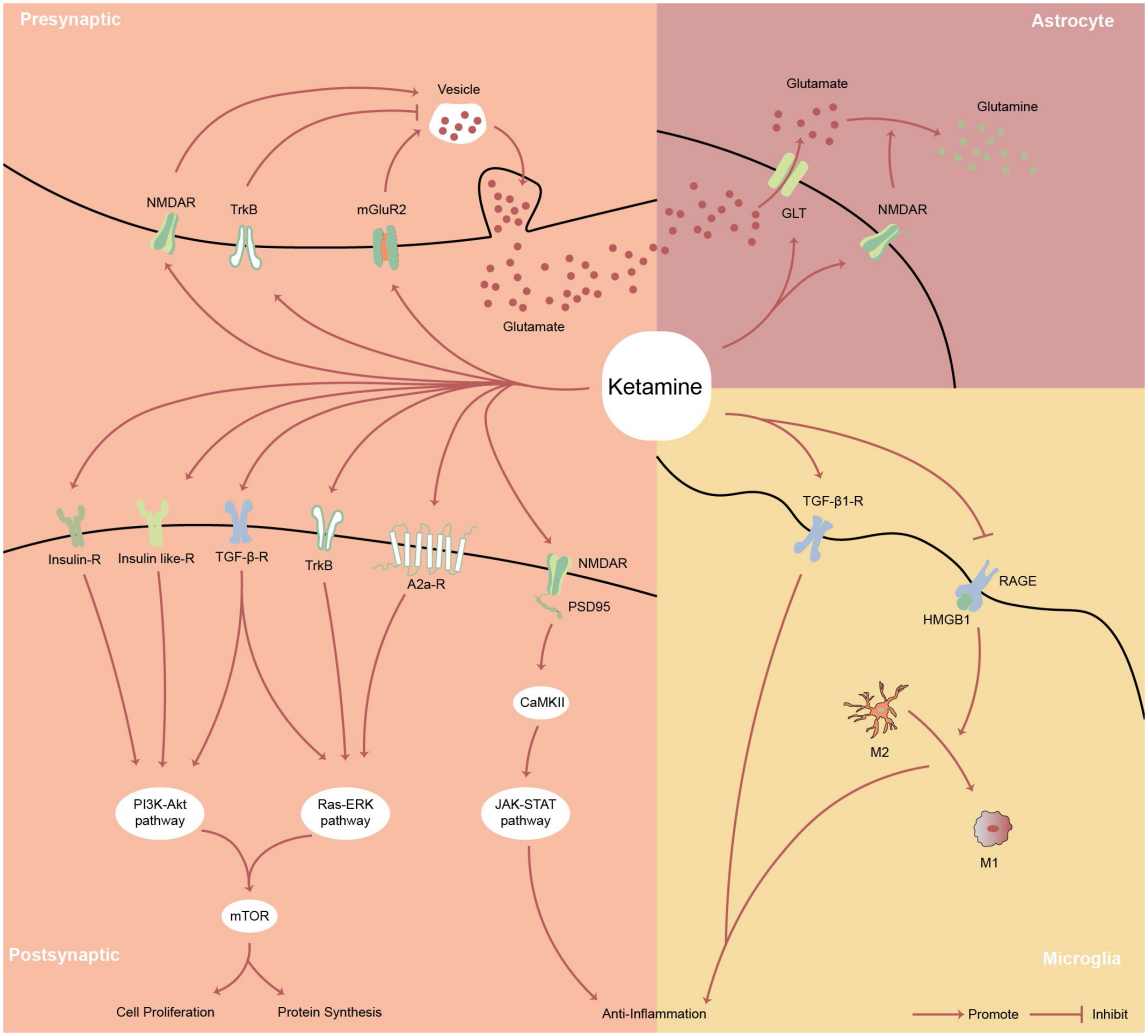
This figure illustrates ketamine's antidepressant mechanisms across presynaptic terminals, postsynaptic regions, astrocytes, and microglia. It shows ketamine modulates glutamate release presynaptically and activates postsynaptic signaling pathways like PI3K-Akt-mTOR and Ras-ERK. In astrocytes, it promotes GLT-1 expression to enhance glutamate uptake. In microglia, ketamine inhibits the NLRP3 inflammasome and shifts polarization to an anti-inflammatory phenotype. Overall, it highlights ketamine's role in restoring synaptic function and suppressing neuroinflammation.

Taken together, these findings suggest that ketamine exerts antidepressant effects not only by enhancing synaptic plasticity but also by stimulating autophagy to suppress neuroinflammation. Nonetheless, while multiple molecular targets have been identified (**Figure 1**), their validation in humans remains challenging. This limitation hinders efforts to optimize ketamine’s molecular structure for maximum antidepressant efficacy while minimizing side effects such as addiction.

This figure illustrates ketamine’s antidepressant mechanisms across presynaptic terminals, postsynaptic regions, astrocytes, and microglia. It shows ketamine modulates glutamate release presynaptically and activates postsynaptic signaling pathways like PI3K-Akt-mTOR and Ras-ERK. In astrocytes, it promotes GLT-1 expression to enhance glutamate uptake. In microglia, ketamine inhibits the NLRP3 inflammasome and shifts polarization to an anti-inflammatory phenotype. Overall, it highlights ketamine’s role in restoring synaptic function and suppressing neuroinflammation.

## 3 Propofol: GABAergic potentiation with antidepressant potential

Propofol is one of the most widely used intravenous sedative anesthetics in clinical practice. In recent years, it has been found that propofol has an unusual effect on the improvement of depressive symptoms (117), particularly in treatment-resistant depression (TRD) (118, 119). Its favorable tolerance profile highlights its potential as a therapeutic option for refractory depression (117), although the precise mechanisms underlying its antidepressant effects remain incompletely understood.

### 3.1 NMDA receptor modulation and cognitive protection

**Figure 2:**
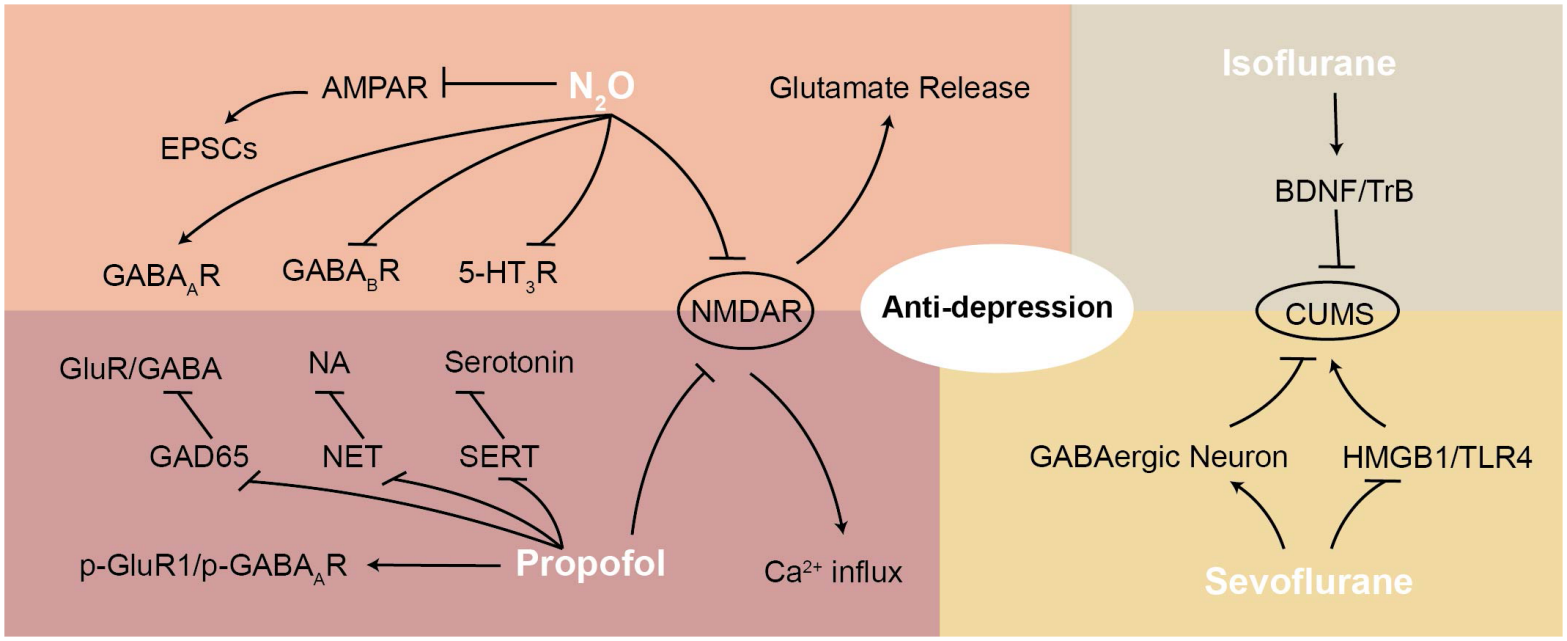
This figure illustrates the mechanisms of various anesthetic agents (N2O, propofol, sevoflurane, and isoflurane) in exerting antidepressant effects, highlighting their interactions with NMDAR and downstream signaling pathways. N2O antagonizes NMDAR and AMPAR, reducing EPSCs and modulating GABA receptors. Propofol enhances GABAergic transmission and regulates p-GluR1/p-GABAAR. Sevoflurane and isoflurane modulate GABAergic neurons and interact with BDNF/TrkB and HMGB1/TLR4 pathways. These mechanisms contribute to anti-depressant effects by restoring synaptic function, regulating neurotransmitter release, and reducing neuroinflammation.

NMDARs are heterotetrameric ion channels typically composed of two GluN1 (NR1) subunits and two GluN2 (NR2A–D) subunits, which are critical regulators of depression-related pathways (120, 121). Although propofol does not appear to affect NMDAR binding affinity or the duration/amplitude of NMDA-activated single-channel openings (**Figure 2**), it reduces the frequency of channel openings in a concentration-dependent and reversible manner, effectively acting as a weak NMDAR antagonist (122, 123). Kingston et al. further reported that propofol reduces phosphorylation of the NR1 subunit (pNR1S897 and pNR1S896) via activation of protein phosphatase 2A. This dephosphorylation attenuates NMDA-induced calcium influx, suggesting inhibitory effects on NMDAR activity, though the direct causal relationship between NR1 dephosphorylation and receptor activity requires further clarification (124).

Interestingly, propofol may also mitigate cognitive impairment associated with electroconvulsive shock (ECS) (125), a procedure with robust antidepressant efficacy but known for memory side effects (126). Synaptic structure plays a key role in learning and memory (127). Combining ECS with propofol reduced ECS-induced activation of NMDARs, thereby lowering long-term potentiation (LTP) and long-term depression (LTD) thresholds, ultimately alleviating memory deficits in stressed rats (128). These findings suggest that ECS combined with propofol may provide synergistic antidepressant benefits while reducing cognitive side effects.

Of note, ECS and propofol appear to achieve antidepressant effects through distinct mechanisms of NMDAR regulation. Excess extrasynaptic glutamate, commonly observed in depressed patients, activates extrasynaptic NMDARs, triggering neurotoxic signaling that leads to synaptic dysfunction and loss (129, 130). ECS reduces extrasynaptic glutamate and upregulates NR2B expression, whereas propofol prevents glutamate-induced NR2B activation, thereby protecting against synaptic damage. (12). This indicates that the antidepressant role of NMDAR modulation may extend beyond simple NR2B antagonism, warranting further mechanistic exploration.

### 3.2 Enhancement of GABA(A) receptor activity

Major depressive disorder (MDD) is associated with reduced brain GABA levels and altered subunit composition of GABA(A) receptors (GABAARs) (131). Propofol enhances GABAAR activity, a property central to its anesthetic effects (132, 133). This enhancement may also contribute to its antidepressant potential. demonstrated that propofol increases phosphorylation of both AMPAR GluR1 and GABAAR subunits in the hippocampus of stressed rats treated with ECS, suggesting coordinated modulation of excitatory and inhibitory balance (134). Moreover, combining low-dose ketamine with propofol stabilized the p-GluR1/p-GABAAR ratio, further enhancing ECS efficacy and alleviating cognitive dysfunctions (134). Luo et al. reported similar findings, showing that propofol mitigates ECS-induced learning and memory deficits by suppressing GAD65 overexpression and restoring the glutamate/GABA balance (135). Collectively, these results suggest that propofol’s antidepressant effects are at least partly mediated by potentiation of GABAAR responses.

### 3.3 Interaction with BDNF and neuroplasticity

BDNF is essential for synaptic plasticity and is strongly implicated in MDD pathophysiology (136–138). Dysregulation of BDNF processing has been linked to depressive states. For example, increased expression of plasminogen activator inhibitor-1 (PAI-1) prevents conversion of proBDNF to mature BDNF (mBDNF), leading to reduced hippocampal BDNF levels in depression models (139, 140).

Evidence suggests that propofol may modulate hippocampal BDNF levels. While some studies report that propofol alone does not significantly alter BDNF expression, its combination with electroconvulsive therapy (ECT) indirectly increases hippocampal BDNF, thereby improving cognition and producing antidepressant effects (140, 141). This highlights a potential synergistic mechanism between propofol and ECT.

### 3.4 Anti-inflammatory properties and oxidative stress control

Inflammation is increasingly recognized as a key factor in depression, with elevated inflammatory markers and acute-phase proteins frequently observed in MDD patients (53, 142). Inflammatory signaling and oxidative stress amplify each other, worsening neurodegeneration and depressive symptoms. Neurosteroids have also been implicated in modulating neuroinflammation in mood and neurodegenerative disorders (143, 144). Propofol exhibits robust anti-inflammatory properties, including inhibition of LPS-induced microglial activation, which may help alleviate inflammation-associated depressive symptoms (145, 146). Mechanistically, propofol reduces inflammation by disrupting metabolic reprogramming (147), suppressing NF-κB signaling (148), modulating adenosine receptors (149), regulating calcium signaling (150), and preventing reactive oxygen species accumulation (148). These findings support propofol’s potential as a therapeutic candidate for depression associated with neuroinflammation.

### 3.5 Effects on monoamine reuptake

Monoamine transporters remain critical targets for antidepressants. Inhibition of the norepinephrine transporter (NET) and serotonin transporter (SERT) increases synaptic levels of NA and 5-HT, thereby alleviating depressive symptoms (151, 152). Zhao and Sun reported that propofol inhibits NET and SERT activity, increasing synaptic concentrations of NA and serotonin (**Figure 2**) (153). However, further studies-particularly those investigating long-term propofol exposure in animal models and humans-are necessary to clarify the clinical relevance of its effects on monoamine transporters in depression.

This figure illustrates the mechanisms of various anesthetic agents (N_2_O, propofol, sevoflurane, and isoflurane) in exerting antidepressant effects, highlighting their interactions with NMDAR and downstream signaling pathways. N_2_O antagonizes NMDAR and AMPAR, reducing EPSCs and modulating GABA receptors. Propofol enhances GABAergic transmission and regulates p-GluR1/p-GABAAR. Sevoflurane and isoflurane modulate GABAergic neurons and interact with BDNF/TrkB and HMGB1/TLR4 pathways. These mechanisms contribute to anti-depressant effects by restoring synaptic function, regulating neurotransmitter release, and reducing neuroinflammation.

## 4 Nitrous oxide: an inhaled antidepressant candidate

Nitrous oxide (N_2_O), commonly used as an inhaled anesthetic and analgesic, has recently drawn attention for its potential antidepressant effects. Inhalation of N_2_O was found to improve depressive symptoms in patients with treatment-resistant depression (TRD). For instance, Nagele conducted two successive research trials that demonstrated N_2_O’s efficacy in improving depressive symptoms, with well-tolerated effects at both 25% and 50% concentrations, lasting up to two weeks. Of these, the 25% concentration had efficacy comparable to 50% but with fewer adverse effects (154, 155). These findings suggest that N_2_O may be a promising adjunctive therapy for TRD, particularly in patients intolerant to conventional treatments.

### 4.1 NMDAR and AMPAR antagonism

The antidepressant effects of NMDA receptor antagonists were initially suggested by Trullas and Skolnick (156). Like ketamine, N_2_O appears to exert antidepressant effects primarily through NMDAR modulation (157, 158). The basolateral amygdala (BLA) plays a crucial role in anesthesia-related amnesia, aversive memory formation, and affective behaviors (159). By antagonizing NMDARs, N_2_O modulates glutamatergic neurotransmission and exerts antidepressant effects in major depressive disorder (160). Although its NMDAR inhibition is weaker (154) and more rapidly reversible (161) than ketamine’s, N_2_O still produces dose-dependent hippocampal neurogenesis and antidepressant effects (162, 163). However, at higher concentrations, N_2_O can cause severe neurotoxic side effects through irreversible vitamin B12 depletion and homocysteine accumulation (164). Moreover, nitric oxide (NO), a metabolite of N_2_O, is neuroprotective at physiological levels but becomes neurotoxic when present in excess (165, 166). For this reason, N_2_O is often co-administered with GABAergic anesthetics to counteract its neurotoxic potential (167).

In addition to NMDAR antagonism, N_2_O also acts as an AMPAR antagonist (**Figure 2**), reducing excitatory postsynaptic currents (EPSCs) and inhibiting action potential-dependent GABA and glutamate release (168). This activity decreases depressive symptoms by modulating AMPAR-mediated signaling while preserving action potential-dependent neurotransmitter release (161, 168).

### 4.2 Modulation of GABAergic transmission

Altered GABA signaling has been implicated in depression, as reduced cerebrospinal fluid GABA levels are observed in patients with major depressive disorder (169). GABA mediates presynaptic inhibition at spinal cord synapses. Mennerick et al. reported that N_2_O slightly prolongs postsynaptic currents via weak enhancement of GABA-A receptor activity (161). Similarly, subsequent experiments confirmed that N_2_O weakly sensitizes GABA-A receptors at postsynaptic sites, enhancing presynaptic inhibition (168). In addition, N_2_O weakly blocks GABA-C receptors and 5-HT3 receptors (170). Clinical 1H-MRS studies have shown that GABA levels normalize in remitted patients, consistent with prior findings of decreased GABA concentrations in depression (171, 172). Thus, N_2_O may alleviate depressive symptoms by weakly enhancing GABA-A receptor function and weakly inhibiting GABA-C receptors (170, 173).

### 4.3 Activation of BDNF-TrkB-mTOR and GSK3β pathways

BDNF levels are significantly reduced in both the brain and serum of patients with MDD (174, 175). Administration of BDNF, either directly into the hippocampus (176) or peripherally (177) produces antidepressant-like effects through activation of the TrkB-AKT-mTOR pathway (175). GSK3β inhibition, regulated by circadian rhythms, further contributes to mTOR activation, which is essential for rapid antidepressant responses (178).

Recent research highlights the role of N_2_O in modulating EEG activity associated with antidepressant effects. In a learned helplessness (LH) model, N_2_O induced a transient phase of cortical excitation followed by a rebound of slow oscillations after cessation of airflow. This rebound phase activated TrkB and GSK3β signaling, suggesting that N_2_O’s fast kinetics may be critical for its rapid antidepressant responses (179). Moreover, mechanistic studies by Liu et al. demonstrated that repeated N_2_O exposure increased burst firing in the mPFC and enhanced BDNF expression in an nNOS-dependent manner (**Figure 2**). These findings indicate that N_2_O may exert antidepressant effects by activating GSK3β signaling and, in turn, upregulating the BDNF-TrkB-AKT-mTOR pathway (111).

### 4.4 Engagement of the endogenous opioid system

The endogenous opioid system-comprising endorphins, enkephalins, dynorphins, and their μ-, δ-, and κ-opioid receptors-plays an important role in stress regulation (180). Several studies suggest that N_2_O primarily targets κ-opioid receptors to mediate analgesic effects, while bypassing μ-opioid receptor activity (181). In addition, N_2_O promotes the release of opioid peptides, such as methionine-enkephalin and β-endorphin, in the periaqueductal gray matter, further contributing to its analgesic effects (182, 183). At subanesthetic doses, N_2_O may preferentially act on central opioid receptors, implicating the opioidergic system in its antidepressant mechanisms. Although the role of opioid signaling in N_2_O’s antidepressant effects remains incompletely defined, it represents an important area for future investigation.

### 4.5 Cerebral vasodilation and vascular effects

Depression is frequently associated with cardiovascular dysfunction. Patients with MDD are at higher risk of new-onset cardiovascular disease, accelerated atherosclerosis, and early vascular aging (184). Experimental models also demonstrate that CUMS induces both depressive behaviors and endothelial dysfunction (185). These may suggest us that depression might be accompanied by effects on blood flow. Neuroimaging studies show that regional cerebral blood flow (rCBF) in the anterior cingulate cortex and dorsal prefrontal cortex is reduced in depressed patients. Increasing rCBF in these regions has been associated with improved mood (186–188). Interestingly, inhaled anesthetics such as N_2_O cause cerebral vasodilation, thereby increasing rCBF (167, 189).This hemodynamic effect may represent an additional mechanism contributing to N_2_O’s antidepressant actions, beyond its effects on neurotransmission and neurotrophic signaling.

## 5 Sevoflurane: inhaled anesthetic with mood-regulating effects

Sevoflurane is a commonly used inhalation anesthetic in clinical practice that has recently demonstrated potential antidepressant properties, particularly in combination with electroconvulsive therapy (ECT). For instance, Guo et al. reported that 2% sevoflurane exposure alleviated depressive-like behavior in a CUMS model by modulating the HMGB1-TLR4 pathway (190), In addition, a clinical case series described significant improvement in a patient with refractory depression following low-dose sevoflurane treatment (191).These findings suggest that sevoflurane may represent a promising adjunctive or alternative therapeutic strategy for depression.

### 5.1 Regulation of GABAergic neurons

Sevoflurane may exert antidepressant effects by modulating GABAergic neurons in the nucleus ambiguus (192). The nucleus ambiguus, which forms part of the dopaminergic circuitry (193), contains GABAergic medium spiny neurons that play a major role in mood regulation (194). However, its effects appear to vary depending on developmental stage and dose. In aged mice, sevoflurane exposure promoted radixin phosphorylation, redistributing anchored 5α-GABAARs toward extrasynaptic sites, which was associated with neurotoxic effects (195). In contrast, in neonates, sevoflurane’s potentiation of GABAAR activity may increase susceptibility to post-anesthetic stress, potentially affecting neurodevelopment (196). These findings indicate that sevoflurane’s actions on GABAergic targets can produce divergent outcomes depending on age and context, in addition to dosage.

### 5.2 Dopaminergic D1 receptor involvement

Evoflurane also influences dopaminergic signaling, particularly through dopamine D1 receptors (D1Rs), which are involved in consciousness and mood regulation. Activation of D1R-expressing neurons in the nucleus accumbens was shown to delay sevoflurane-induced anesthesia and accelerate recovery, suggesting that D1R neurons regulate anesthesia-related alterations in consciousness (197). Furthermore, uncoupling of D1R and D2R has been implicated in the rapid relief of depressive symptoms (198). Supporting this, Noori et al. demonstrated that nucleus accumbens D1-D2 receptor heteromers may play a role in mitigating postpartum depression (199). Taken together, these findings suggest that sevoflurane may exert antidepressant effects, at least in part, through D1R-related modulation.

Although numerous animal studies have demonstrated sevoflurane’s antidepressant efficacy (**Figure 2**), its independent effectiveness and long-term safety in patients remain uncertain, with only limited clinical cases reported to date (191, 200). Given its established anesthetic profile and preliminary evidence of antidepressant potential, further investigations into sevoflurane’s safety, efficacy, and mechanisms are warranted before its clinical application in depression can be fully realized.

## 6 Isoflurane: revisiting a classic agent for depression therapy

Isoflurane, an inhaled anesthetic, has been studied for antidepressant potential since the 1980s, with early evidence showing rapid effects: Langer et al. first reported its therapeutic value in 1985, and later demonstrated in a controlled trial that isoflurane combined with electroconvulsive therapy (ECT) improved psychometric outcomes in severely depressed women (201, 202). Isoflurane, an inhaled general anesthetic and structural isomer of enflurane, has been investigated for its psychotherapeutic potential since the mid-1980s. Early clinical evidence suggested that isoflurane could produce rapid antidepressant effects: Langer et al. first reported its therapeutic value in 1985, and later demonstrated in a controlled trial that isoflurane combined with electroconvulsive therapy (ECT) improved psychometric outcomes in severely depressed women (201, 202). Mechanistically, isoflurane shares several pathways with other anesthetic antidepressants. It has been shown to rapidly alleviate CUMS-induced depressive symptoms through activation of the BDNF-TrkB pathway (**Figure 2**) (203). Isoflurane also selectively inhibits mitochondrial complex I and presynaptic excitatory signaling, leading to decreased presynaptic ATP levels and suppression of synaptic vesicle cycling (204). This mitochondrial inhibition, a hallmark of volatile anesthetics, may contribute to its antidepressant effects by reducing excitatory drive. Consistently, dose-dependent cortical EEG suppression under isoflurane anesthesia has been linked to modulation of learned helplessness behaviors (205). In addition, isoflurane enhances GABAergic transmission, promotes neuroplasticity, and modulates parvalbumin interneurons via TrkB and mTOR signaling pathways, further supporting its potential antidepressant mechanisms (206). Unlike ketamine, isoflurane does not produce psychomimetic side effects or carry a significant risk of abuse, making it an attractive candidate for clinical repurposing.

Although isoflurane is no longer widely used as a first-line anesthetic in modern practice, its distinctive pharmacological profile, safety in controlled use, and evidence of antidepressant efficacy highlight its potential as a therapeutic alternative for treatment-resistant depression. Further clinical studies are needed to establish its long-term safety, efficacy, and optimal therapeutic regimen.

## 7 Summary

### 7.1 Glutamatergic and GABAergic regulation

Across all anesthetic agents reviewed, restoration of the excitatory–inhibitory (E/I) balance emerges as a central antidepressant mechanism. Ketamine and nitrous oxide (N^2^O) primarily act as NMDAR antagonists, leading to compensatory AMPAR activation, enhanced synaptic plasticity, and rapid restoration of excitatory signaling. In contrast, propofol, sevoflurane, and isoflurane potentiate GABA-A receptor function, strengthening inhibitory neurotransmission and normalizing cortical hyperexcitability. Collectively, these mechanisms converge on synaptic remodeling through coordinated modulation of NMDAR, AMPAR, and GABAAR signaling, ultimately reducing excitotoxicity and re-establishing functional connectivity within prefrontal–limbic circuits.

### 7.2 Neurotrophic pathways

All five anesthetic agents enhance neurotrophic signaling, particularly through BDNF-TrkB and mTOR cascades that promote synaptogenesis and neuronal resilience. Ketamine induces a rapid increase in BDNF expression and activates TrkB-mTOR via CaMKII and PI3K/Akt pathways, underpinning its fast-acting antidepressant effects. N_2_O and isoflurane also stimulate TrkB-mTOR signaling, whereas propofol indirectly elevates BDNF levels—especially when used in conjunction with electroconvulsive therapy (ECT). Sevoflurane exerts dual effects by enhancing neurotrophic signaling while suppressing inflammation through HMGB1-TLR4 inhibition. Together, these findings highlight the BDNF-TrkB-mTOR axis as a shared molecular denominator across anesthetic-based antidepressants.

### 7.3 Anti-inflammatory and HPA axis modulation

Chronic inflammation and hyperactivation of the hypothalamic–pituitary–adrenal (HPA) axis are central contributors to depression pathophysiology. Ketamine and propofol alleviate depressive behaviors by suppressing NLRP3 inflammasome activation and reducing pro-inflammatory cytokine release. Sevoflurane inhibits the HMGB1-TLR4 pathway, while isoflurane and N_2_O attenuate microglial activation and glial-driven inflammation. Several agents also normalize corticosterone or cortisol levels, reflecting restoration of HPA axis homeostasis. Collectively, these anti-inflammatory and neuroendocrine regulatory effects suggest that anesthetic agents act not only on neurotransmission but also on systemic stress and immune responses.

### 7.4 Mitochondrial and autophagy regulation

Mitochondrial protection and enhanced autophagy further contribute to the neuroprotective and antidepressant properties of anesthetic agents. Ketamine and isoflurane stimulate autophagy-mediated synaptic protein turnover, supporting synaptic renewal and energy balance. Propofol mitigates oxidative stress and prevents mitochondrial dysfunction through NF-κB inhibition and antioxidative mechanisms. These effects collectively maintain neuronal integrity and promote cellular homeostasis, providing an additional layer of protection against stress-induced neurodegeneration (**Table 1**).

**Table 1:** Primary molecular targets and signaling pathways of anesthetic antidepressants.

Agent	Primary targets	Key pathways involved	Dominant neurobiological effects
Ketamine	NMDAR (GluN2B), AMPAR, mGluR2	BDNF-TrkB-mTOR, ERK-CREB, NLRP3 inhibition	Rapid synaptogenesis, reduced inflammation
Propofol	GABAAR, NMDA (NR1 dephosphorylation)	BDNF-ERK, NF-κB suppression, antioxidant response	Enhanced GABAergic tone, neuroprotection
N_2_O	NMDAR, AMPAR, GABAAR (weak)	TrkB-GSK3β-mTOR, nNOS activation	Increased neurogenesis, cortical excitation–inhibition balance
Sevoflurane	GABAAR, D1R, HMGB1-TLR4	BDNF-TrkB, inflammatory suppression	Anti-inflammatory, mood stabilization
Isoflurane	GABAAR, TrkB, mTOR	BDNF-TrkB-mTOR, mitochondrial regulation	Enhanced plasticity, reduced excitatory drive

### 7.5 Integration of preclinical and clinical findings

Overall, ketamine remains the most extensively validated anesthetic with both preclinical and clinical evidence of antidepressant efficacy. Propofol and N_2_O show consistent but smaller-scale evidence, while sevoflurane and isoflurane demonstrate promising mechanistic overlap but limited human data. Together, these findings indicate a shared pattern of synaptic restoration, neurotrophic activation, and anti-inflammatory modulation across anesthetic classes. (**Table 2****and****3**).

**Table 2:** Preclinical and clinical evidence for each agent.

Agent	Preclinical evidence	Clinical evidence	Level of evidence
Ketamine	Robust evidence from CUMS, CRS, and CSDS models; multiple molecular targets validated	>40 RCTs and meta-analyses show rapid efficacy in TRD	High
Propofol	Animal models show modulation of GABAAR and BDNF pathways	Case reports and small RCTs in TRD or ECT settings	Moderate
N_2_O	Demonstrated neurogenesis and TrkB activation in rodents	Two randomized crossover trials and several pilot studies	Moderate
Sevoflurane	Anti-inflammatory and GABAergic effects in mice	Limited clinical case reports	Low–Moderate
Isoflurane	Antidepressant-like effects and TrkB activation in animals	Historical human studies (1980s-1990s); small modern series	Low–Moderate

**Table 3:** Clinical outcomes and safety profiles.

Agent	Onset & duration of effect	Reported side effects	Safety/tolerability
Ketamine	Rapid (hours), lasts 1–7 days	Dissociation, BP elevation, potential abuse	Well-tolerated with monitoring
Propofol	Rapid but transient mood improvement	Hypotension, respiratory depression (dose-dependent)	Safe under clinical supervision
N2O	Within hours, sustained up to 2 weeks	Nausea, mild dizziness; B_12_ depletion with chronic use	Generally safe; avoid prolonged exposure
Sevoflurane	Onset within 24 h (single exposure in rodents); human duration unknown	Cognitive impairment at high doses	Good short-term tolerability
Isoflurane	Rapid improvement in mood and cognition	Hypotension, anesthesia-related effects	Safe in controlled settings

## 8 Challenges and future perspectives

### 8.1 Safety and ethical considerations

Although anesthetic-based antidepressants, particularly ketamine and its enantiomer esketamine, demonstrate rapid and robust antidepressant efficacy, their clinical implementation raises important safety and ethical considerations. First, the potential for misuse and dependence cannot be overlooked. Clinical and epidemiological evidence indicates that repeated or non-medical ketamine exposure is associated with abuse liability, cognitive impairment, and urological toxicity, emphasizing the need for stringent prescribing limits, addiction risk assessment, and follow-up monitoring (207). Second, concerns regarding neurodevelopmental safety in pediatric and adolescent populations remain substantial. Both preclinical and clinical studies have suggested that early or repeated exposure to general anesthetics may disrupt synaptic development and neurocognitive maturation (208, 209). Therefore, anesthetic-based interventions in younger populations should be limited to controlled research settings with rigorous long-term cognitive and behavioral follow-up. Third, in older adults, anesthetic exposure has been associated with postoperative cognitive dysfunction (POCD) and possible long-term neurocognitive decline, particularly in individuals with vascular or neurodegenerative comorbidities (210). Accordingly, any clinical use of anesthetic antidepressants in elderly patients should include baseline cognitive screening, peri-treatment monitoring, and post-treatment neuropsychological assessment.

### 8.2 Translational challenges

Translating anesthetic agents from perioperative or procedural use to mainstream psychiatric care presents significant regulatory, clinical, and socioeconomic challenges. From a regulatory perspective, obtaining approval for psychiatric indications requires extensive Phase III randomized controlled trials to demonstrate sustained efficacy and long-term safety, as exemplified by esketamine’s FDA approval process in 2019. Moreover, approved use typically entails controlled settings, qualified personnel, and post-administration observation. Clinically, effective integration of anesthetic-based antidepressants demands interdisciplinary collaboration between anesthesiology and psychiatry, establishment of standardized protocols for patient screening, administration, and monitoring, and incorporation of psychosocial support into treatment workflows. Economically, limited infrastructure and monitoring requirements increase per-session cost and may restrict accessibility, particularly in resource-limited healthcare systems. Addressing these translational barriers will require coordinated efforts in regulatory alignment, healthcare delivery reform, and cost-effectiveness evaluation.

### 8.3 Need for long-term follow-up and biomarker development

Most current clinical trials focus on short-term outcomes—typically spanning several days to a few weeks—thereby leaving the long-term safety, durability of response, and relapse patterns of anesthetic-based antidepressants largely unknown. Future research should prioritize longitudinal follow-up studies extending from 6 to 24 months, systematically assessing sustained efficacy, adverse events (including potential dependence), and functional outcomes such as cognitive and social recovery. In parallel, there is an urgent need to develop and validate biomarkers—including neuroimaging parameters, electrophysiological signatures, genetic and inflammatory markers—to identify patients most likely to benefit from anesthetic antidepressants with minimal risk. Such precision approaches could ultimately inform personalized psychiatry and rational therapeutic selection (211).

### 8.4 Future research priorities

To advance the field responsibly, future research should emphasize:

(1) Large-scale, multicenter randomized controlled trials (RCTs) directly comparing different anesthetic agents (e.g., intravenous ketamine, nitrous oxide, propofol) with standard antidepressants and placebo across both short- and long-term intervals.

(2) Head-to-head comparative studies examining relative efficacy and safety among anesthetic compounds and versus other rapid-acting agents, such as esketamine nasal spray.

(3) Optimization of dosing and administration paradigms, including dose-response studies, alternative routes (intravenous, inhaled, intranasal), and maintenance strategies.

(4) Combination therapy research, exploring potential synergy between anesthetic antidepressants and psychotherapy, repetitive transcranial magnetic stimulation (rTMS), or maintenance pharmacotherapy.

Finally, as these novel therapeutics redefine treatment-resistant depression management, they may also transform the paradigm of psychiatric care—from symptomatic relief toward precision psychiatry grounded in molecular profiling, longitudinal monitoring, and interdisciplinary collaboration.

## 9 Conclusion

Anesthetics once regarded solely as agents for sedation and analgesia are now recognized as potential rapid-acting antidepressants. Evidence from both clinical and preclinical studies highlights their ability to alleviate depressive symptoms through diverse yet interconnected mechanisms. These include modulation of glutamatergic and GABAergic transmission, regulation of monoaminergic and opioid systems, enhancement of neurotrophic signaling pathways such as BDNF-TrkB-mTOR, suppression of neuroinflammation, and restoration of neural plasticity. Agents such as ketamine, propofol, nitrous oxide, sevoflurane, and isoflurane demonstrate distinct pharmacological profiles but converge on common molecular and circuit-level mechanisms that underlie mood regulation.

Despite encouraging findings, significant challenges remain. The long-term safety of repeated anesthetic exposure, potential neurotoxic effects in vulnerable populations, and the risk-benefit balance relative to established antidepressants require careful consideration. Furthermore, most clinical evidence to date derives from small trials or case series, emphasizing the need for larger, well-designed studies to confirm efficacy, optimize dosing regimens, and identify patient subgroups most likely to benefit.

Looking ahead, the exploration of anesthetics as antidepressants offers a unique opportunity to bridge anesthesiology and psychiatry. Continued mechanistic research may not only guide the rational repurposing of existing agents but also inspire the development of novel therapeutics that retain antidepressant efficacy while minimizing adverse effects. With careful clinical translation, anesthetic-based interventions may expand the therapeutic armamentarium for treatment-resistant depression and contribute to a new era of precision neuropsychiatric care.

## References

[1] Santomauro DF Mantilla Herrera AM Shadid J Zheng P Ashbaugh C Pigott DM . Global prevalence and burden of depressive and anxiety disorders in 204 countries and territories in 2020 due to the COVID-19 pandemic. Lancet. (2021) 398:1700–12. doi: 10.1016/S0140-6736(21)02143-7, PMID: 34634250 PMC8500697

[2] Chen YA Fan T Toma CL Scherr S . International students’ psychosocial well-being and social media use at the onset of the COVID-19 pandemic: A latent profile analysis. Comput Hum Behav. (2022) 137:107409. doi: 10.1016/j.chb.2022.107409, PMID: 35936989 PMC9338770

[3] Guo J Zhao Y Wang J Fang L Liu S Luo X . The associations among the stress symptoms, depressive symptoms, anxiety symptoms and insomnia symptoms in depressed patients after the first COVID-19 outbreak was initially controlled in China: A prospective cohort study. J Affect Disord. (2022) 314:253–8. doi: 10.1016/j.jad.2022.07.021, PMID: 35872249 PMC9304076

[4] Berman RM Cappiello A Anand A Oren DA Heninger GR Charney DS . Antidepressant effects of ketamine in depressed patients. Biol Psychiatry. (2000) 47:351–4. doi: 10.1016/s0006-3223(99)00230-9, PMID: 10686270

[5] Breault MS Orguc S Kwon O Kang GH Tseng B Schreier DR . Anesthetics as treatments for depression: Clinical insights and underlying mechanisms. Annu Rev Neurosci. (2025) 48:103–24. doi: 10.1146/annurev-neuro-112723-062031, PMID: 39971384

[6] d’Andrea G Cavallotto C Pettorruso M Lorenzo GD Carullo R De Berardis D . Effectiveness of repeated Esketamine nasal spray administration on anhedonic symptoms in treatment-resistant bipolar and unipolar depression: A secondary analysis from the REAL-ESK study group. Psychiatry Res. (2025) 352:116655. doi: 10.1016/j.psychres.2025.116655, PMID: 40865291

[7] Rosso G d’Andrea G Barlati S Di Nicola M Andriola I Marcatili M . Esketamine treatment trajectory of patients with treatment-resistant depression in the mid and long-term run: data from REAL-ESK study group. Curr Neuropharmacol. (2025) 23:612–9. doi: 10.2174/011570159X337670241029062524, PMID: 39810448 PMC12163464

[8] Price RB Kissel N Baumeister A Rohac R Woody ML Ballard ED . International pooled patient-level meta-analysis of ketamine infusion for depression: In search of clinical moderators. Mol Psychiatry. (2022) 27:5096–112. doi: 10.1038/s41380-022-01757-7, PMID: 36071111 PMC9763119

[9] Di Nicola M Pepe M d’Andrea G Marcelli I Pettorruso M Andriola I . Patient experience with intranasal esketamine in treatment-resistant depression: insights from a multicentric italian study (REAL-ESKperience). J Personalized Med. (2025) 15:161. doi: 10.3390/jpm15040161, PMID: 40278340 PMC12029048

[10] Gastaldon C Raschi E Kane JM Barbui C Schoretsanitis G . Post-marketing safety concerns with esketamine: A disproportionality analysis of spontaneous reports submitted to the FDA adverse event reporting system. Psychother Psychosom. (2021) 90:41–8. doi: 10.1159/000510703, PMID: 32854103

[11] Cui L Li S Wang S Wu X Liu Y Yu W . Major depressive disorder: Hypothesis, mechanism, prevention and treatment. Signal Transduc Target Ther. (2024) 9:30. doi: 10.1038/s41392-024-01738-y, PMID: 38331979 PMC10853571

[12] Le Meur K Galante M Angulo MC Audinat E . Tonic activation of NMDA receptors by ambient glutamate of non-synaptic origin in the rat hippocampus. J Physiol. (2007) 580:373–83. doi: 10.1113/jphysiol.2006.123570, PMID: 17185337 PMC2075557

[13] Hardingham GE Bading H . Synaptic versus extrasynaptic NMDA receptor signalling: implications for neurodegenerative disorders. Nat Rev Neurosci. (2010) 11:682–96. doi: 10.1038/nrn2911, PMID: 20842175 PMC2948541

[14] Li N Liu R-J Dwyer JM Banasr M Lee B Son H . Glutamate N-methyl-D-aspartate receptor antagonists rapidly reverse behavioral and synaptic deficits caused by chronic stress exposure. Biol Psychiatry. (2011) 69:754–61. doi: 10.1016/j.biopsych.2010.12.015, PMID: 21292242 PMC3068225

[15] Acero VP Cribas ES Browne KD Rivellini O Burrell JC O’Donnell JC . Bedside to bench: The outlook for psychedelic research. Front Pharmacol. (2023) 14:1240295. doi: 10.3389/fphar.2023.1240295, PMID: 37869749 PMC10588653

[16] Irie M Hata Y Takeuchi M Ichtchenko K Toyoda A Hirao K . Binding of neuroligins to PSD-95. Science. (1997) 277:1511–5. doi: 10.1126/science.277.5331.1511, PMID: 9278515

[17] Gegelashvili G Dehnes Y Danbolt NC Schousboe A . The high-affinity glutamate transporters GLT1, GLAST, and EAAT4 are regulated via different signalling mechanisms. Neurochem Int. (2000) 37:163–70. doi: 10.1016/s0197-0186(00)00019-x, PMID: 10812201

[18] Nădășan IK Hancu G . Psychotherapy, pharmacotherapy, and their combination in the treatment of major depressive disorder: How well are we making use of available therapies? Acta Marisiens - Seria Med. (2023) 69:244–51. doi: 10.2478/amma-2023-0042

[19] Liu W-X Wang J Xie Z-M Xu N Zhang G-F Jia M . Regulation of glutamate transporter 1 via BDNF-TrkB signaling plays a role in the anti-apoptotic and antidepressant effects of ketamine in chronic unpredictable stress model of depression. Psychopharmacol (Berl). (2016) 233:405–15. doi: 10.1007/s00213-015-4128-2, PMID: 26514555

[20] Dosemeci A Makusky AJ Jankowska-Stephens E Yang X Slotta DJ Markey SP . Composition of the synaptic PSD-95 complex. Mol Cell Proteomics. (2007) 6:1749–60. doi: 10.1074/mcp.M700040-MCP200, PMID: 17623647 PMC2096750

[21] Compans B Camus C Kallergi E Sposini S Martineau M Butler C . NMDAR-dependent long-term depression is associated with increased short term plasticity through autophagy mediated loss of PSD-95. Nat Commun. (2021) 12:2849. doi: 10.1038/s41467-021-23133-9, PMID: 33990590 PMC8121912

[22] Zhang W-J Wang H-H Lv Y-D Liu C-C Sun W-Y Tian L-J . Downregulation of egr-1 expression level via gluN2B underlies the antidepressant effects of ketamine in a chronic unpredictable stress animal model of depression. Neuroscience. (2018) 372:38–45. doi: 10.1016/j.neuroscience.2017.12.045, PMID: 29294341

[23] Bemben MA Shipman SL Hirai T Herring BE Li Y Badger JD . CaMKII phosphorylation of neuroligin-1 regulates excitatory synapses. Nat Neurosci. (2014) 17:56–64. doi: 10.1038/nn.3601, PMID: 24336150 PMC3943352

[24] Edem EE Anyanwu C-KC Nebo KE Akinluyi ET Fafure AA Ishola AO . Ketamine abrogates sensorimotor deficits and cytokine dysregulation in a chronic unpredictable mild stress model of depression. Psychopharmacol (Berl). (2022) 239:185–200. doi: 10.1007/s00213-021-06021-4, PMID: 34792632

[25] Li J-M Liu L-L Su W-J Wang B Zhang T Zhang Y . Ketamine may exert antidepressant effects via suppressing NLRP3 inflammasome to upregulate AMPA receptors. Neuropharmacology. (2019) 146:149–53. doi: 10.1016/j.neuropharm.2018.11.022, PMID: 30496753

[26] Camargo A Dalmagro AP Wolin IAV Kaster MP Rodrigues ALS . The resilient phenotype elicited by ketamine against inflammatory stressors-induced depressive-like behavior is associated with NLRP3-driven signaling pathway. J Psychiatr Res. (2021) 144:118–28. doi: 10.1016/j.jpsychires.2021.09.057, PMID: 34619490

[27] Pandya CD Hoda N Crider A Peter D Kutiyanawalla A Kumar S . Transglutaminase 2 overexpression induces depressive-like behavior and impaired TrkB signaling in mice. Mol Psychiatry. (2017) 22:745–53. doi: 10.1038/mp.2016.145, PMID: 27620841 PMC5348279

[28] Zanos P Highland JN Stewart BW Georgiou P Jenne CE Lovett J . (2R,6R)-hydroxynorketamine exerts mGlu2 receptor-dependent antidepressant actions. Proc Natl Acad Sci U.S.A. (2019) 116:6441–50. doi: 10.1073/pnas.1819540116, PMID: 30867285 PMC6442605

[29] Nasca C Zelli D Bigio B Piccinin S Scaccianoce S Nisticò R . Stress dynamically regulates behavior and glutamatergic gene expression in hippocampus by opening a window of epigenetic plasticity. Proc Natl Acad Sci U.S.A. (2015) 112:14960–5. doi: 10.1073/pnas.1516016112, PMID: 26627246 PMC4672825

[30] Pałucha-Poniewiera A Podkowa K Rafało-Ulińska A . The group II mGlu receptor antagonist LY341495 induces a rapid antidepressant-like effect and enhances the effect of ketamine in the chronic unpredictable mild stress model of depression in C57BL/6J mice. Prog Neuropsychopharmacol Biol Psychiatry. (2021) 109:110239. doi: 10.1016/j.pnpbp.2020.110239, PMID: 33400944

[31] Witkin JM Mitchell SN Wafford KA Carter G Gilmour G Li J . Comparative effects of LY3020371, a potent and selective metabotropic glutamate (mGlu) 2/3 receptor antagonist, and ketamine, a noncompetitive N-methyl-d-aspartate receptor antagonist in rodents: evidence supporting the use of mGlu2/3 antagonists, for the treatment of depression. J Pharmacol Exp Ther. (2017) 361:68–86. doi: 10.1124/jpet.116.238121, PMID: 28138040

[32] AANS S Baxter B Campbell BCV Carpenter JS Cognard C Dippel D . Multisociety consensus quality improvement revised consensus statement for endovascular therapy of acute ischemic stroke. Int J Stroke. (2018) 13:612–32. doi: 10.1177/1747493018778713, PMID: 29786478

[33] Fukumoto K Iijima M Chaki S The antidepressant effects of an mGlu2/3 receptor antagonist and ketamine require AMPA receptor stimulation in the mPFC and subsequent activation of the 5-HT neurons in the DRN. Neuropsychopharmacology. (2016) 41:1046–1056. doi: 10.1038/npp.2015.233, PMID: 26245499 PMC4748429

[34] Autry AE Adachi M Nosyreva E Na ES Los MF Cheng P . NMDA receptor blockade at rest triggers rapid behavioural antidepressant responses. Nature. (2011) 475:91–5. doi: 10.1038/nature10130, PMID: 21677641 PMC3172695

[35] Seo MK Lee JA Jeong S Seog D-H Lee JG Park SW . Effects of chronic LY341495 on hippocampal mTORC1 signaling in mice with chronic unpredictable stress-induced depression. Int J Mol Sci. (2022) 23:6416. doi: 10.3390/ijms23126416, PMID: 35742857 PMC9224204

[36] Hashimoto K Zhao M Zhu T Wang X Yang J . Ketamine and its two enantiomers in anesthesiology and psychiatry: A historical review and future directions. J Anesth Trans Med. (2024) 3:65–75. doi: 10.1016/j.jatmed.2024.07.001 PMC13001759 41929866

[37] Maciag D Hughes J O’Dwyer G Pride Y Stockmeier CA Sanacora G . Reduced density of calbindin immunoreactive GABAergic neurons in the occipital cortex in major depression: relevance to neuroimaging studies. Biol Psychiatry. (2010) 67:465–70. doi: 10.1016/j.biopsych.2009.10.027, PMID: 20004363 PMC2823848

[38] Lucido MJ Dunlop BW . Emerging medications for treatment-resistant depression: A review with perspective on mechanisms and challenges. Brain Sci. (2025) 15:161. doi: 10.3390/brainsci15020161, PMID: 40002494 PMC11853532

[39] Kim CS Johnston D . Antidepressant effects of (S)-ketamine through a reduction of hyperpolarization-activated current ih. iScience. (2020) 23:101239. doi: 10.1016/j.isci.2020.101239, PMID: 32629607 PMC7322259

[40] Shi W Wei X Wang X Du S Liu W Song J . Perineuronal nets protect long-term memory by limiting activity-dependent inhibition from parvalbumin interneurons. Proc Natl Acad Sci U.S.A. (2019) 116:27063–73. doi: 10.1073/pnas.1902680116, PMID: 31843906 PMC6936502

[41] Gerhard DM Pothula S Liu R-J Wu M Li X-Y Girgenti MJ . GABA interneurons are the cellular trigger for ketamine’s rapid antidepressant actions. J Clin Invest. (2020) 130:1336–49. doi: 10.1172/JCI130808, PMID: 31743111 PMC7269589

[42] Yu Z Chen N Hu D Chen W Yuan Y Meng S . Decreased density of perineuronal net in prelimbic cortex is linked to depressive-like behavior in young-aged rats. Front Mol Neurosci. (2020) 13:4. doi: 10.3389/fnmol.2020.00004, PMID: 32116542 PMC7025547

[43] Picard N Takesian AE Fagiolini M Hensch TK . NMDA 2A receptors in parvalbumin cells mediate sex-specific rapid ketamine response on cortical activity. Mol Psychiatry. (2019) 24:828–38. doi: 10.1038/s41380-018-0341-9, PMID: 30696941 PMC6756203

[44] Su T Lu Y Fu C Geng Y Chen Y . GluN2A mediates ketamine-induced rapid antidepressant-like responses. Nat Neurosci. (2023) 26:1751–61. doi: 10.1038/s41593-023-01436-y, PMID: 37709995

[45] Yu Z Han Y Hu D Chen N Zhang Z Chen W . Neurocan regulates vulnerability to stress and the anti-depressant effect of ketamine in adolescent rats. Mol Psychiatry. (2022) 27:2522–32. doi: 10.1038/s41380-022-01495-w, PMID: 35264728

[46] Hu W Zhang M Czéh B Flügge G Zhang W . Stress impairs GABAergic network function in the hippocampus by activating nongenomic glucocorticoid receptors and affecting the integrity of the parvalbumin-expressing neuronal network. Neuropsychopharmacology. (2010) 35:1693–707. doi: 10.1038/npp.2010.31, PMID: 20357756 PMC3055473

[47] Yin C Xu M Zong Z . Advances in the prevalence and treatment of depression for adolescents: A review. Front Pharmacol. (2025) 16:1574574. doi: 10.3389/fphar.2025.1574574, PMID: 40406494 PMC12096414

[48] Lamers F Vogelzangs N Merikangas KR de Jonge P Beekman ATF Penninx BWJH . Evidence for a differential role of HPA-axis function, inflammation and metabolic syndrome in melancholic versus atypical depression. Mol Psychiatry. (2013) 18:692–9. doi: 10.1038/mp.2012.144, PMID: 23089630

[49] Ouyang X Wang Z Luo M Wang M Liu X Chen J . Ketamine ameliorates depressive-like behaviors in mice through increasing glucose uptake regulated by the ERK/GLUT3 signaling pathway. Sci Rep. (2021) 11:18181. doi: 10.1038/s41598-021-97758-7, PMID: 34518608 PMC8437933

[50] Luo Y Yu Y Zhang M He H Fan N . Chronic administration of ketamine induces cognitive deterioration by restraining synaptic signaling. Mol Psychiatry. (2021) 26:4702–18. doi: 10.1038/s41380-020-0793-6, PMID: 32488127

[51] Lv D Chen Y Shen M Liu X Zhang Y Xu J . Mechanisms underlying the rapid-acting antidepressant-like effects of neuropeptide VGF (non-acronymic) C-terminal peptide TLQP-62. Neuropharmacology. (2018) 143:317–26. doi: 10.1016/j.neuropharm.2018.09.046, PMID: 30291938

[52] Recinella L Chiavaroli A Orlando G Ferrante C Veschi S Cama A . Effects of growth hormone-releasing hormone receptor antagonist MIA-602 in mice with emotional disorders: a potential treatment for PTSD. Mol Psychiatry. (2021) 26:7465–74. doi: 10.1038/s41380-021-01228-5, PMID: 34331008

[53] Anisman H Hayley S . Inflammatory factors contribute to depression and its comorbid conditions. Sci Signal. (2012) 5:pe45. doi: 10.1126/scisignal.2003579, PMID: 23033537

[54] Xie Z-M Wang X-M Xu N Wang J Pan W Tang X-H . Alterations in the inflammatory cytokines and brain-derived neurotrophic factor contribute to depression-like phenotype after spared nerve injury: improvement by ketamine. Sci Rep. (2017) 7:3124. doi: 10.1038/s41598-017-03590-3, PMID: 28600519 PMC5466642

[55] Wang T Weng H Zhou H Yang Z Tian Z Xi B . Esketamine alleviates postoperative depression-like behavior through anti-inflammatory actions in mouse prefrontal cortex. J Affect Disord. (2022) 307:97–107. doi: 10.1016/j.jad.2022.03.072, PMID: 35378150

[56] Grassi D Franz H Vezzali R Bovio P Heidrich S Dehghanian F . Neuronal activity, TGFβ-signaling and unpredictable chronic stress modulate transcription of gadd45 family members and DNA methylation in the hippocampus. Cereb Cortex. (2017) 27:4166–81. doi: 10.1093/cercor/bhx095, PMID: 28444170

[57] Vivien D Ali C . Transforming growth factor-beta signalling in brain disorders. Cytokine Growth Factor Rev. (2006) 17:121–8. doi: 10.1016/j.cytogfr.2005.09.011, PMID: 16271500

[58] Lee K-M Kim Y-K . The role of IL-12 and TGF-beta1 in the pathophysiology of major depressive disorder. Int Immunopharmacol. (2006) 6:1298–304. doi: 10.1016/j.intimp.2006.03.015, PMID: 16782542

[59] Rush G O’Donovan A Nagle L Conway C McCrohan A O’Farrelly C . Alteration of immune markers in a group of melancholic depressed patients and their response to electroconvulsive therapy. J Affect Disord. (2016) 205:60–8. doi: 10.1016/j.jad.2016.06.035, PMID: 27414954 PMC5291160

[60] Caraci F Gulisano W Guida CA Impellizzeri AAR Drago F Puzzo D . A key role for TGF-β1 in hippocampal synaptic plasticity and memory. Sci Rep. (2015) 5:11252. doi: 10.1038/srep11252, PMID: 26059637 PMC4462026

[61] Depino AM LucChina L Pitossi F . Early and adult hippocampal TGF-β1 overexpression have opposite effects on behavior. Brain Behav Immun. (2011) 25:1582–91. doi: 10.1016/j.bbi.2011.05.007, PMID: 21640817

[62] Zhang J Qu Y Chang L Pu Y Hashimoto K . (R)-ketamine rapidly ameliorates the decreased spine density in the medial prefrontal cortex and hippocampus of susceptible mice after chronic social defeat stress. Int J Neuropsychopharmacol. (2019) 22:675–9. doi: 10.1093/ijnp/pyz048, PMID: 31504547 PMC6822137

[63] Yao W Cao Q Luo S He L Yang C Chen J . Microglial ERK-NRBP1-CREB-BDNF signaling in sustained antidepressant actions of (R)-ketamine. Mol Psychiatry. (2022) 27:1618–29. doi: 10.1038/s41380-021-01377-7, PMID: 34819637 PMC9095473

[64] Fukushima T Liu R-Y Byrne JH . Transforming growth factor-beta2 modulates synaptic efficacy and plasticity and induces phosphorylation of CREB in hippocampal neurons. Hippocampus. (2007) 17:5–9. doi: 10.1002/hipo.20243, PMID: 17094084

[65] Zhang K Yang C Chang L Sakamoto A Suzuki T Fujita Y . Essential role of microglial transforming growth factor-β1 in antidepressant actions of (R)-ketamine and the novel antidepressant TGF-β1. Transl Psychiatry. (2020) 10:32. doi: 10.1038/s41398-020-0733-x, PMID: 32066676 PMC7026089

[66] Chinthapalli K . Cortisol levels predict depression in teenage boys, study shows. BMJ. (2014) 348:g1654. doi: 10.1136/bmj.g1654, PMID: 24558204

[67] Busillo JM Azzam KM Cidlowski JA . Glucocorticoids sensitize the innate immune system through regulation of the NLRP3 inflammasome. J Biol Chem. (2011) 286:38703–13. doi: 10.1074/jbc.M111.275370, PMID: 21940629 PMC3207479

[68] Xian X Cai L-L Li Y Wang R-C Xu Y-H Chen Y-J . Neuron secrete exosomes containing miR-9-5p to promote polarization of M1 microglia in depression. J Nanobiotechnol. (2022) 20:122. doi: 10.1186/s12951-022-01332-w, PMID: 35264203 PMC8905830

[69] Ali T Rahman SU Hao Q Li W Liu Z Ali Shah F . Melatonin prevents neuroinflammation and relieves depression by attenuating autophagy impairment through FOXO3a regulation. J Pineal Res. (2020) 69:e12667. doi: 10.1111/jpi.12667, PMID: 32375205

[70] Liu Y Hu P Zheng Z Zhong D Xie W Tang Z . Photoresponsive vaccine-like CAR-M system with high-efficiency central immune regulation for inflammation-related depression. Adv Mater. (2022) 34:e2108525. doi: 10.1002/adma.202108525, PMID: 34897839

[71] LeGates TA Altimus CM Wang H Lee H-K Yang S Zhao H . Aberrant light directly impairs mood and learning through melanopsin-expressing neurons. Nature. (2012) 491:594–8. doi: 10.1038/nature11673, PMID: 23151476 PMC3549331

[72] Liu R-J Fuchikami M Dwyer JM Lepack AE Duman RS Aghajanian GK . GSK-3 inhibition potentiates the synaptogenic and antidepressant-like effects of subthreshold doses of ketamine. Neuropsychopharmacology. (2013) 38:2268–77. doi: 10.1038/npp.2013.128, PMID: 23680942 PMC3773678

[73] Yang C Ali T Li A Gao R Yu X Li S . Ketamine reverses chronic corticosterone-induced behavioral deficits and hippocampal synaptic dysfunction by regulating eIF4E/BDNF signaling. Neuropharmacology. (2024) 261:110156. doi: 10.1016/j.neuropharm.2024.110156, PMID: 39326783

[74] Zhu J Hu Z Han X Wang D Jiang Q Ding J . Dopamine D2 receptor restricts astrocytic NLRP3 inflammasome activation via enhancing the interaction of β-arrestin2 and NLRP3. Cell Death Differ. (2018) 25:2037–49. doi: 10.1038/s41418-018-0127-2, PMID: 29786071 PMC6219479

[75] Song A-Q Gao B Fan J-J Zhu Y-J Zhou J Wang Y-L . NLRP1 inflammasome contributes to chronic stress-induced depressive-like behaviors in mice. J Neuroinflamm. (2020) 17:178. doi: 10.1186/s12974-020-01848-8, PMID: 32513185 PMC7281929

[76] Alcocer-Gómez E Casas-Barquero N Williams MR Romero-Guillena SL Cañadas-Lozano D Bullón P . Antidepressants induce autophagy dependent-NLRP3-inflammasome inhibition in Major depressive disorder. Pharmacol Res. (2017) 121:114–21. doi: 10.1016/j.phrs.2017.04.028, PMID: 28465217

[77] Yang Y Xing M-J Li Y Zhang H-F Yuan T-F Peng D-H . Reduced NLRP3 inflammasome expression in the brain is associated with stress resilience. Psychoneuroendocrinology. (2021) 128:105211. doi: 10.1016/j.psyneuen.2021.105211, PMID: 33812228

[78] Lyu D Wang F Zhang M Yang W Huang H Huang Q . Ketamine induces rapid antidepressant effects via the autophagy-NLRP3 inflammasome pathway. Psychopharmacol (Berl). (2022) 239:3201–12. doi: 10.1007/s00213-022-06201-w, PMID: 35925279

[79] Jiang B Wang H Wang J-L Wang Y-J Zhu Q Wang C-N . Hippocampal salt-inducible kinase 2 plays a role in depression via the CREB-regulated transcription coactivator 1-cAMP response element binding-brain-derived neurotrophic factor pathway. Biol Psychiatry. (2019) 85:650–66. doi: 10.1016/j.biopsych.2018.10.004, PMID: 30503507

[80] Wang Y Liu L Gu J-H Wang C-N Guan W Liu Y . Salt-inducible kinase 1-CREB-regulated transcription coactivator 1 signalling in the paraventricular nucleus of the hypothalamus plays a role in depression by regulating the hypothalamic-pituitary-adrenal axis. Mol Psychiatry. (2024) 29:1660–70. doi: 10.1038/s41380-022-01881-4, PMID: 36434056

[81] Wei Z Zhang K Zhou Q Huang M Xu T Dong J . Differential mechanisms underlying antidepressant responses of ketamine and imipramine. CNS Neurol Disord Drug Targets. (2017) 16:846–53. doi: 10.2174/1871527316666170428123248, PMID: 28462695

[82] Meylan EM Breuillaud L Seredenina T Magistretti PJ Halfon O Luthi-Carter R . Involvement of the agmatinergic system in the depressive-like phenotype of the Crtc1 knockout mouse model of depression. Transl Psychiatry. (2016) 6:e852. doi: 10.1038/tp.2016.116, PMID: 27404284 PMC5545706

[83] Fabbri C Hosak L Mössner R Giegling I Mandelli L Bellivier F . Consensus paper of the WFSBP Task Force on Genetics: Genetics, epigenetics and gene expression markers of major depressive disorder and antidepressant response. World J Biol Psychiatry. (2017) 18:5–28. doi: 10.1080/15622975.2016.1208843, PMID: 27603714

[84] Maglio LE Noriega-Prieto JA Maroto IB Martin-Cortecero J Muñoz-Callejas A Callejo-Móstoles M . IGF-1 facilitates extinction of conditioned fear. Elife. (2021) 10:e67267. doi: 10.7554/eLife.67267, PMID: 33792539 PMC8043742

[85] Ota KT Andres W Lewis DA Stockmeier CA Duman RS . BICC1 expression is elevated in depressed subjects and contributes to depressive behavior in rodents. Neuropsychopharmacology. (2015) 40:711–8. doi: 10.1038/npp.2014.227, PMID: 25178406 PMC4289959

[86] Shen M Lv D Liu X Wang C . ERK/mTOR signaling may underlying the antidepressant actions of rapastinel in mice. Trans Psychiatry. (2022) 12:522. doi: 10.1038/s41398-022-02290-5, PMID: 36550125 PMC9780240

[87] Jaine R Kvizhinadze G Nair N Blakely T . Cost-effectiveness of a low-dose computed tomography screening programme for lung cancer in New Zealand. Lung Cancer. (2018) 124:233–40. doi: 10.1016/j.lungcan.2018.08.004, PMID: 30268467

[88] Lewis CM Ng MY Butler AW Cohen-Woods S Uher R Pirlo K . Genome-wide association study of major recurrent depression in the U.K. population. Am J Psychiatry. (2010) 167:949–57. doi: 10.1176/appi.ajp.2010.09091380, PMID: 20516156

[89] Alder J Thakker-Varia S Bangasser DA Kuroiwa M Plummer MR Shors TJ . Brain-derived neurotrophic factor-induced gene expression reveals novel actions of VGF in hippocampal synaptic plasticity. J Neurosci. (2003) 23:10800–8. doi: 10.1523/JNEUROSCI.23-34-10800.2003, PMID: 14645472 PMC3374594

[90] Hunsberger JG Newton SS Bennett AH Duman CH Russell DS Salton SR . Antidepressant actions of the exercise-regulated gene VGF. Nat Med. (2007) 13:1476–82. doi: 10.1038/nm1669, PMID: 18059283

[91] Jiang C Lin WJ Sadahiro M Labonté B Menard C Pfau ML . VGF function in depression and antidepressant efficacy. Mol Psychiatry. (2018) 23:1632–1642. doi: 10.1038/mp.2017.233, PMID: 29158577 PMC5962361

[92] Shen M Lv D Liu X Li S Chen Y Zhang Y . Essential roles of neuropeptide VGF regulated TrkB/mTOR/BICC1 signaling and phosphorylation of AMPA receptor subunit GluA1 in the rapid antidepressant-like actions of ketamine in mice. Brain Res Bull. (2018) 143:58–65. doi: 10.1016/j.brainresbull.2018.10.004, PMID: 30316917

[93] Camargo A Torrá ACNC Dalmagro AP Valverde AP Kouba BR Fraga DB . Prophylactic efficacy of ketamine, but not the low-trapping NMDA receptor antagonist AZD6765, against stress-induced maladaptive behavior and 4E-BP1-related synaptic protein synthesis impairment. Prog Neuropsychopharmacol Biol Psychiatry. (2022) 115:110509. doi: 10.1016/j.pnpbp.2022.110509, PMID: 35033626

[94] Hodes GE Pfau ML Purushothaman I Ahn HF Golden SA Christoffel DJ . Sex differences in nucleus accumbens transcriptome profiles associated with susceptibility versus resilience to subchronic variable stress. J Neurosci. (2015) 35:16362–76. doi: 10.1523/JNEUROSCI.1392-15.2015, PMID: 26674863 PMC4679819

[95] Sato H Fukutani Y Yamamoto Y Tatara E Takemoto M Shimamura K . Thalamus-derived molecules promote survival and dendritic growth of developing cortical neurons. J Neurosci. (2012) 32:15388–402. doi: 10.1523/JNEUROSCI.0293-12.2012, PMID: 23115177 PMC6621586

[96] Seo J-S Wei J Qin L Kim Y Yan Z Greengard P . Cellular and molecular basis for stress-induced depression. Mol Psychiatry. (2017) 22:1440–7. doi: 10.1038/mp.2016.118, PMID: 27457815 PMC5269558

[97] Thakker-Varia S Krol JJ Nettleton J Bilimoria PM Bangasser DA Shors TJ . The neuropeptide VGF produces antidepressant-like behavioral effects and enhances proliferation in the hippocampus. J Neurosci. (2007) 27:12156–67. doi: 10.1523/JNEUROSCI.1898-07.2007, PMID: 17989282 PMC3363962

[98] Thakker-Varia S Behnke J Doobin D Dalal V Thakkar K Khadim F . VGF (TLQP-62)-induced neurogenesis targets early phase neural progenitor cells in the adult hippocampus and requires glutamate and BDNF signaling. Stem Cell Res. (2014) 12:762–77. doi: 10.1016/j.scr.2014.03.005, PMID: 24747217 PMC4991619

[99] Zhang F Luo J Zhu X . Ketamine ameliorates depressive-like behaviors by tPA-mediated conversion of proBDNF to mBDNF in the hippocampus of stressed rats. Psychiatry Res. (2018) 269:646–51. doi: 10.1016/j.psychres.2018.08.075, PMID: 30216916

[100] Jiang C Lin W-J Labonté B Tamminga CA Turecki G Nestler EJ . VGF and its C-terminal peptide TLQP-62 in ventromedial prefrontal cortex regulate depression-related behaviors and the response to ketamine. Neuropsychopharmacology. (2019) 44:971–81. doi: 10.1038/s41386-018-0277-4, PMID: 30504797 PMC6462025

[101] Zeng J Ji Y Luan F Hu J Rui Y Liu Y . Xiaoyaosan ethyl acetate fraction alleviates depression-like behaviors in CUMS mice by promoting hippocampal neurogenesis via modulating the IGF-1Rβ/PI3K/Akt signaling pathway. J Ethnopharmacol. (2022) 288:115005. doi: 10.1016/j.jep.2022.115005, PMID: 35051601

[102] Malberg JE Platt B Rizzo SJ Ring RH Lucki I Schechter LE . Increasing the levels of insulin-like growth factor-I by an IGF binding protein inhibitor produces anxiolytic and antidepressant-like effects. Neuropsychopharmacology. (2007) 32:2360–2368. doi: 10.1038/sj.npp.1301358, PMID: 17342171

[103] Deyama S Kondo M Shimada S Kaneda K . IGF-1 release in the medial prefrontal cortex mediates the rapid and sustained antidepressant-like actions of ketamine. Transl Psychiatry. (2022) 12:178. doi: 10.1038/s41398-022-01943-9, PMID: 35577782 PMC9110717

[104] Li Y Chen Y Gao X Zhang Z . The behavioral deficits and cognitive impairment are correlated with decreased IGF-II and ERK in depressed mice induced by chronic unpredictable stress. Int J Neurosci. (2017) 127:1096–103. doi: 10.1080/00207454.2017.1337014, PMID: 28562144

[105] Luo Y-W Xu Y Cao W-Y Zhong X-L Duan J Wang X-Q . Insulin-like growth factor 2 mitigates depressive behavior in a rat model of chronic stress. Neuropharmacology. (2015) 89:318–24. doi: 10.1016/j.neuropharm.2014.10.011, PMID: 25446675

[106] Grieco SF Cheng Y Eldar-Finkelman H Jope RS Beurel E . Up-regulation of insulin-like growth factor 2 by ketamine requires glycogen synthase kinase-3 inhibition. Prog Neuropsychopharmacol Biol Psychiatry. (2017) 72:49–54. doi: 10.1016/j.pnpbp.2016.08.008, PMID: 27542584 PMC5061618

[107] Grossert A Mehrjardi NZ Bailey SJ Lindsay MA Hescheler J Šarić T . Ketamine Increases Proliferation of Human iPSC-Derived Neuronal Progenitor Cells via Insulin-Like Growth Factor 2 and Independent of the NMDA Receptor. Cells. (2019) 8:1139. doi: 10.3390/cells8101139, PMID: 31554266 PMC6830315

[108] Jung S Choe S Woo H Jeong H An H-K Moon H . Autophagic death of neural stem cells mediates chronic stress-induced decline of adult hippocampal neurogenesis and cognitive deficits. Autophagy. (2020) 16:512–30. doi: 10.1080/15548627.2019.1630222, PMID: 31234698 PMC6999625

[109] Zhang M Lyu D Wang F Shi S Wang M Yang W . Ketamine may exert rapid antidepressant effects through modulation of neuroplasticity, autophagy, and ferroptosis in the habenular nucleus. Neuroscience. (2022) 506:29–37. doi: 10.1016/j.neuroscience.2022.10.015, PMID: 36280022

[110] Cao H Zuo C Huang Y Zhu L Zhao J Yang Y . Hippocampal proteomic analysis reveals activation of necroptosis and ferroptosis in a mouse model of chronic unpredictable mild stress-induced depression. Behav Brain Res. (2021) 407:113261. doi: 10.1016/j.bbr.2021.113261, PMID: 33775778

[111] Liu W Li Q Ye B Cao H Shen F Xu Z . Repeated nitrous oxide exposure exerts antidepressant-like effects through neuronal nitric oxide synthase activation in the medial prefrontal cortex. Front Psychiatry. (2020) 11:837. doi: 10.3389/fpsyt.2020.00837, PMID: 33088274 PMC7495238

[112] Kallergi E Daskalaki AD Kolaxi A Camus C Ioannou E Mercaldo V . Dendritic autophagy degrades postsynaptic proteins and is required for long-term synaptic depression in mice. Nat Commun. (2022) 13:680. doi: 10.1038/s41467-022-28301-z, PMID: 35115539 PMC8814153

[113] Tang M Liu T Jiang P Dang R . The interaction between autophagy and neuroinflammation in major depressive disorder: From pathophysiology to therapeutic implications. Pharmacol Res. (2021) 168:105586. doi: 10.1016/j.phrs.2021.105586, PMID: 33812005

[114] Lu J-J Wu P-F He J-G Li Y-K Long L-H Yao X-P . BNIP3L/NIX-mediated mitophagy alleviates passive stress-coping behaviors induced by tumor necrosis factor-α. Mol Psychiatry. (2023) 28:5062–76. doi: 10.1038/s41380-023-02008-z, PMID: 36914810

[115] Li X Li Y Zhao J Li L Wang Y Zhang Y . Administration of ketamine causes autophagy and apoptosis in the rat fetal hippocampus and in PC12 cells. Front Cell Neurosci. (2018) 12:21. doi: 10.3389/fncel.2018.00021, PMID: 29456493 PMC5801406

[116] Wu M Zhao L Wang Y Guo Q An Q Geng J . Ketamine regulates the autophagy flux and polarization of microglia through the HMGB1-RAGE axis and exerts antidepressant effects in mice. J Neuropathol Exp Neurol. (2022) 81:931–42. doi: 10.1093/jnen/nlac035, PMID: 35582883

[117] Mickey BJ White AT Arp AM Leonardi K Torres MM Larson AL . Propofol for treatment-resistant depression: A pilot study. Int J Neuropsychopharmacol. (2018) 21:1079–89. doi: 10.1093/ijnp/pyy085, PMID: 30260415 PMC6276046

[118] Zhong X He H Zhang C Wang Z Jiang M Li Q . Mood and neuropsychological effects of different doses of ketamine in electroconvulsive therapy for treatment-resistant depression. J Affect Disord. (2016) 201:124–30. doi: 10.1016/j.jad.2016.05.011, PMID: 27208499

[119] Zheng W He M Gu L-M Lao G-H Wang D-F Mai J-X . Early improvement as a predictor of final remission in patients with treatment-resistant depression receiving electroconvulsive therapy with ketofol anesthesia. J Affect Disord. (2022) 310:223–7. doi: 10.1016/j.jad.2022.05.027, PMID: 35550826

[120] Chou T-H Epstein M Michalski K Fine E Biggin PC Furukawa H . Structural insights into binding of therapeutic channel blockers in NMDA receptors. Nat Struct Mol Biol. (2022) 29:507–18. doi: 10.1038/s41594-022-00772-0, PMID: 35637422 PMC10075384

[121] Ruffini G Castaldo F Lopez-Sola E Sanchez-Todo R Vohryzek J . The algorithmic agent perspective and computational neuropsychiatry: From etiology to advanced therapy in major depressive disorder. Entropy. (2024) 26:953. doi: 10.3390/e26110953, PMID: 39593898 PMC11592617

[122] Orser BA Bertlik M Wang LY MacDonald JF . Inhibition by propofol (2,6 di-isopropylphenol) of the N-methyl-D-aspartate subtype of glutamate receptor in cultured hippocampal neurones. Br J Pharmacol. (1995) 116:1761–8. doi: 10.1111/j.1476-5381.1995.tb16660.x, PMID: 8528557 PMC1909100

[123] Yamakura T Sakimura K Shimoji K Mishina M . Effects of propofol on various AMPA-, kainate- and NMDA-selective glutamate receptor channels expressed in Xenopus oocytes. Neurosci Lett. (1995) 188:187–90. doi: 10.1016/0304-3940(95)11431-u, PMID: 7609905

[124] Kingston S Mao L Yang L Arora A Fibuch EE Wang JQ . Propofol inhibits phosphorylation of N-methyl-D-aspartate receptor NR1 subunits in neurons. Anesthesiology. (2006) 104:763–9. doi: 10.1097/00000542-200604000-00021, PMID: 16571972

[125] Luo J Min S Wei K Cao J Wang B Li P . Behavioral and molecular responses to electroconvulsive shock differ between genetic and environmental rat models of depression. Psychiatry Res. (2015) 226:451–60. doi: 10.1016/j.psychres.2014.12.068, PMID: 25708608

[126] Sackeim HA Prudic J Fuller R Keilp J Lavori PW Olfson M . The cognitive effects of electroconvulsive therapy in community settings. Neuropsychopharmacology. (2007) 32:244–54. doi: 10.1038/sj.npp.1301180, PMID: 16936712

[127] Cai DJ Aharoni D Shuman T Shobe J Biane J Song W . A shared neural ensemble links distinct contextual memories encoded close in time. Nature. (2016) 534:115–8. doi: 10.1038/nature17955, PMID: 27251287 PMC5063500

[128] Ren L Hao X Min S Deng J Chen Q Chen H . Anesthetics alleviate learning and memory impairment induced by electroconvulsive shock by regulation of NMDA receptor-mediated metaplasticity in depressive rats. Neurobiol Learn Mem. (2018) 155:65–77. doi: 10.1016/j.nlm.2018.06.013, PMID: 29953948

[129] Massey PV Johnson BE Moult PR Auberson YP Brown MW Molnar E . Differential roles of NR2A and NR2B-containing NMDA receptors in cortical long-term potentiation and long-term depression. J Neurosci. (2004) 24:7821–8. doi: 10.1523/JNEUROSCI.1697-04.2004, PMID: 15356193 PMC6729941

[130] Li A-H Bu S Wang L Liang A-M Luo H-Y . Impact of propofol and sevoflurane anesthesia on cognition and emotion in gastric cancer patients undergoing radical resection. World J Gastrointestin Oncol. (2024) 16:79–89. doi: 10.4251/wjgo.v16.i1.79, PMID: 38292851 PMC10824106

[131] Luscher B Shen Q Sahir N . The GABAergic deficit hypothesis of major depressive disorder. Mol Psychiatry. (2011) 16:383–406. doi: 10.1038/mp.2010.120, PMID: 21079608 PMC3412149

[132] Jayakar SS Zhou X Chiara DC Dostalova Z Savechenkov PY Bruzik KS . Multiple propofol-binding sites in a γ-aminobutyric acid type A receptor (GABAAR) identified using a photoreactive propofol analog. J Biol Chem. (2014) 289:27456–68. doi: 10.1074/jbc.M114.581728, PMID: 25086038 PMC4183786

[133] Tadler SC Jones KG Lybbert C Huang JC Jawish R Solzbacher D . Propofol for treatment resistant depression: A randomized controlled trial. medRxiv: Preprint Serv Health Sci. (2023). doi: 10.1101/2023.09.12.23294678, PMID: 37745479 PMC10516089

[134] Chen J Peng L-H Luo J Liu L Lv F Li P . Effects of low-dose ketamine combined with propofol on phosphorylation of AMPA receptor GluR1 subunit and GABAA receptor in hippocampus of stressed rats receiving electroconvulsive shock. J ECT. (2015) 31:50–6. doi: 10.1097/YCT.0000000000000148, PMID: 24831997

[135] Luo J Min S Wei K Li P Dong J Liu Y-F . Propofol protects against impairment of learning-memory and imbalance of hippocampal Glu/GABA induced by electroconvulsive shock in depressed rats. J Anesth. (2011) 25:657–65. doi: 10.1007/s00540-011-1199-z, PMID: 21769668

[136] Sen S Duman R Sanacora G . Serum brain-derived neurotrophic factor, depression, and antidepressant medications: meta-analyses and implications. Biol Psychiatry. (2008) 64:527–32. doi: 10.1016/j.biopsych.2008.05.005, PMID: 18571629 PMC2597158

[137] Guilloux J-P Douillard-Guilloux G Kota R Wang X Gardier AM Martinowich K . Molecular evidence for BDNF- and GABA-related dysfunctions in the amygdala of female subjects with major depression. Mol Psychiatry. (2012) 17:1130–42. doi: 10.1038/mp.2011.113, PMID: 21912391 PMC3237836

[138] Duman RS Deyama S Fogaça MV . Role of BDNF in the pathophysiology and treatment of depression: Activity-dependent effects distinguish rapid-acting antidepressants. Eur J Neurosci. (2021) 53:126–39. doi: 10.1111/ejn.14630, PMID: 31811669 PMC7274898

[139] Chandler WL Alessi MC Aillaud MF Henderson P Vague P Juhan-Vague I . Clearance of tissue plasminogen activator (TPA) and TPA/plasminogen activator inhibitor type 1 (PAI-1) complex: relationship to elevated TPA antigen in patients with high PAI-1 activity levels. Circulation. (1997) 96:761–8. doi: 10.1161/01.cir.96.3.761, PMID: 9264480

[140] Zhang F Luo J Min S Ren L Qin P . Propofol alleviates electroconvulsive shock-induced memory impairment by modulating proBDNF/mBDNF ratio in depressive rats. Brain Res. (2016) 1642:43–50. doi: 10.1016/j.brainres.2016.03.020, PMID: 27017958

[141] Sun Z Jia L Shi D He Y Ren Y . Deep brain stimulation improved depressive-like behaviors and hippocampal synapse deficits by activating the BDNF/mTOR signaling pathway. Behav Brain Res. (2022) 419:113709.doi:10.1016/j.bbr.2021.113709. doi: 10.1016/j.bbr.2021.113709, PMID: 34890598

[142] Marx W Penninx BW Solmi M Furukawa TA Firth J Carvalho AF . Major depressive disorder. Nat Rev Dis Primers. (2023) 9:44. doi: 10.1038/s41572-023-00454-1, PMID: 37620370

[143] Zhou R Yazdi AS Menu P Tschopp J . A role for mitochondria in NLRP3 inflammasome activation. Nature. (2011) 469:221–5. doi: 10.1038/nature09663, PMID: 21124315

[144] Singhal M Modi N Bansal L Abraham J Mehta I Ravi A . The emerging role of neurosteroids: Novel drugs brexanalone, sepranolone, zuranolone, and ganaxolone in mood and neurological disorders. Cureus. (2024) 16:e65866.doi:10.7759/cureus.65866. doi: 10.7759/cureus.65866, PMID: 39219949 PMC11364262

[145] Liu J Ai P Sun Y Yang X Li C Liu Y . Propofol Inhibits Microglial Activation via miR-106b/Pi3k/Akt Axis. Front Cell Neurosci. (2021) 15:768364. doi: 10.3389/fncel.2021.768364, PMID: 34776870 PMC8581742

[146] Xiao X Hou Y Yu W Qi S . Propofol ameliorates microglia activation by targeting microRNA-221/222-IRF2 axis. J Immunol Res. (2021) 2021:3101146. doi: 10.1155/2021/3101146, PMID: 34423051 PMC8373515

[147] Guan S Sun L Wang X Huang X Luo T . Propofol inhibits neuroinflammation and metabolic reprogramming in microglia *in vitro* and in *vivo*. Front Pharmacol. (2023) 14:1161810. doi: 10.3389/fphar.2023.1161810, PMID: 37383725 PMC10293632

[148] Cheng L Chen Z Wang L Lan Y Zheng L Wu F . Propofol partially attenuates complete freund’s adjuvant-induced neuroinflammation through inhibition of the ERK1/2/NF-κB pathway. J Cell Biochem. (2019) 120:9400–8. doi: 10.1002/jcb.28215, PMID: 30536812

[149] Yu H Wang X Kang F Chen Z Meng Y Dai M . Propofol attenuates inflammatory damage on neurons following cerebral infarction by inhibiting excessive activation of microglia. Int J Mol Med. (2019) 43:452–60. doi: 10.3892/ijmm.2018.3974, PMID: 30431058

[150] Lu Y Gu Y Ding X Wang J Chen J Miao C . Intracellular Ca2+ homeostasis and JAK1/STAT3 pathway are involved in the protective effect of propofol on BV2 microglia against hypoxia-induced inflammation and apoptosis. PloS One. (2017) 12:e0178098. doi: 10.1371/journal.pone.0178098, PMID: 28542400 PMC5441598

[151] Andersen J Stuhr-Hansen N Zachariassen L Toubro S Hansen SMR Eildal JNN . Molecular determinants for selective recognition of antidepressants in the human serotonin and norepinephrine transporters. Proc Natl Acad Sci U.S.A. (2011) 108:12137–42. doi: 10.1073/pnas.1103060108, PMID: 21730142 PMC3141962

[152] Marshe VS Maciukiewicz M Rej S Tiwari AK Sibille E Blumberger DM . Norepinephrine transporter gene variants and remission from depression with venlafaxine treatment in older adults. Am J Psychiatry. (2017) 174:468–75. doi: 10.1176/appi.ajp.2016.16050617, PMID: 28068779

[153] Zhao Y Sun L . Antidepressants modulate the *in vitro* inhibitory effects of propofol and ketamine on norepinephrine and serotonin transporter function. J Clin Neurosci. (2008) 15:1264–9. doi: 10.1016/j.jocn.2007.11.007, PMID: 18815045 PMC2605271

[154] Nagele P Duma A Kopec M Gebara MA Parsoei A Walker M . Nitrous oxide for treatment-resistant major depression: A proof-of-concept trial. Biol Psychiatry. (2015) 78:10–8. doi: 10.1016/j.biopsych.2014.11.016, PMID: 25577164

[155] Nagele P Palanca BJ Gott B Brown F Barnes L Nguyen T . A phase 2 trial of inhaled nitrous oxide for treatment-resistant major depression. Sci Transl Med. (2021) 13:eabe1376. doi: 10.1126/scitranslmed.abe1376, PMID: 34108247

[156] Trullas R Skolnick P . Functional antagonists at the NMDA receptor complex exhibit antidepressant actions. Eur J Pharmacol. (1990) 185:1–10. doi: 10.1016/0014-2999(90)90204-j, PMID: 2171955

[157] Walsh K Das RK Kamboj SK . The subjective response to nitrous oxide is a potential pharmaco-endophenotype for alcohol use disorder: A preliminary study with heavy drinkers. Int J Neuropsychopharmacol. (2017) 20:346–50. doi: 10.1093/ijnp/pyw063, PMID: 27401180 PMC5409036

[158] Kamboj SK Zhao H Troebinger L Piazza G Cawley E Hennessy V . Rewarding subjective effects of the NMDAR antagonist nitrous oxide (Laughing gas) are moderated by impulsivity and depressive symptoms in healthy volunteers. Int J Neuropsychopharmacol. (2021) 24:551–61. doi: 10.1093/ijnp/pyab009, PMID: 33667308 PMC8299821

[159] Tye KM Prakash R Kim S-Y Fenno LE Grosenick L Zarabi H . Amygdala circuitry mediating reversible and bidirectional control of anxiety. Nature. (2011) 471:358–62. doi: 10.1038/nature09820, PMID: 21389985 PMC3154022

[160] Myles PS Kulkarni J Kasza J Wallace S Deng C Turbić A . Antidepressant effects of nitrous oxide in major depressive disorder: A phase 2b randomized clinical trial. Biol Psychiatry Global Open Sci. (2025) 5:100504. doi: 10.1016/j.bpsgos.2025.100504, PMID: 40503328 PMC12155549

[161] Mennerick S Jevtovic-Todorovic V Todorovic SM Shen W Olney JW Zorumski CF . Effect of nitrous oxide on excitatory and inhibitory synaptic transmission in hippocampal cultures. J Neurosci. (1998) 18:9716–26. doi: 10.1523/JNEUROSCI.18-23-09716.1998, PMID: 9822732 PMC6793274

[162] Nacher J Alonso-Llosa G Rosell DR McEwen BS . NMDA receptor antagonist treatment increases the production of new neurons in the aged rat hippocampus. Neurobiol Aging. (2003) 24:273–84. doi: 10.1016/s0197-4580(02)00096-9, PMID: 12498961

[163] Chamaa F Bahmad HF Makkawi A-K Chalhoub RM Al-Chaer ED Bikhazi GB . Nitrous oxide induces prominent cell proliferation in adult rat hippocampal dentate gyrus. Front Cell Neurosci. (2018) 12:135. doi: 10.3389/fncel.2018.00135, PMID: 29867368 PMC5967150

[164] Temple C Horowitz BZ . Nitrous oxide abuse induced subacute combined degeneration despite patient initiated B12 supplementation. Clin Toxicol (Phila). (2022) 60:872–5. doi: 10.1080/15563650.2022.2046772, PMID: 35253567

[165] Abraini JH David HN Lemaire M . Potentially neuroprotective and therapeutic properties of nitrous oxide and xenon. Ann N Y Acad Sci. (2005) 1053:289–300. doi: 10.1196/annals.1344.025, PMID: 16179534

[166] Cichon J Joseph TT Lu X Wasilczuk AZ Kelz MB Mennerick SJ . Nitrous oxide activates layer 5 prefrontal neurons via SK2 channel inhibition for antidepressant effect. Nat Commun. (2025) 16:2999. doi: 10.1038/s41467-025-57951-y, PMID: 40180931 PMC11968965

[167] Jevtović-Todorović V Todorović SM Mennerick S Powell S Dikranian K Benshoff N . Nitrous oxide (laughing gas) is an NMDA antagonist, neuroprotectant and neurotoxin. Nat Med. (1998) 4:460–3. doi: 10.1038/nm0498-460, PMID: 9546794

[168] Izumi Y Hsu F-F Conway CR Nagele P Mennerick SJ Zorumski CF . Nitrous oxide, a rapid antidepressant, has ketamine-like effects on excitatory transmission in the adult hippocampus. Biol Psychiatry. (2022) 92:964–72. doi: 10.1016/j.biopsych.2022.06.016, PMID: 36050137 PMC10107749

[169] Feyissa AM Chandran A Stockmeier CA Karolewicz B . Reduced levels of NR2A and NR2B subunits of NMDA receptor and PSD-95 in the prefrontal cortex in major depression. Prog Neuropsychopharmacol Biol Psychiatry. (2009) 33:70–5. doi: 10.1016/j.pnpbp.2008.10.005, PMID: 18992785 PMC2655629

[170] Yamakura T Harris RA . Effects of gaseous anesthetics nitrous oxide and xenon on ligand-gated ion channels. Comparison with isoflurane and ethanol. Anesthesiology. (2000) 93:1095–101. doi: 10.1097/00000542-200010000-00034, PMID: 11020766

[171] Fogaça MV Wu M Li C Li X-Y Picciotto MR Duman RS . Inhibition of GABA interneurons in the mPFC is sufficient and necessary for rapid antidepressant responses. Mol Psychiatry. (2021) 26:3277–91. doi: 10.1038/s41380-020-00916-y, PMID: 33070149 PMC8052382

[172] Ritter C Buchmann A Müller ST Volleberg M Haynes M Ghisleni C . Evaluation of prefrontal γ-aminobutyric acid and glutamate levels in individuals with major depressive disorder using proton magnetic resonance spectroscopy. JAMA Psychiatry. (2022) 79:1209–16. doi: 10.1001/jamapsychiatry.2022.3384, PMID: 36260322 PMC9582968

[173] Fuchs T Jefferson SJ Hooper A Yee P-H Maguire J Luscher B . Disinhibition of somatostatin-positive GABAergic interneurons results in an anxiolytic and antidepressant-like brain state. Mol Psychiatry. (2017) 22:920–30. doi: 10.1038/mp.2016.188, PMID: 27821870 PMC5422144

[174] Tripp A Oh H Guilloux J-P Martinowich K Lewis DA Sibille E . Brain-derived neurotrophic factor signaling and subgenual anterior cingulate cortex dysfunction in major depressive disorder. Am J Psychiatry. (2012) 169:1194–202. doi: 10.1176/appi.ajp.2012.12020248, PMID: 23128924 PMC3638149

[175] Lee J Lee KH Kim SH Han JY Hong S-B Cho S-C . Early changes of serum BDNF and SSRI response in adolescents with major depressive disorder. J Affect Disord. (2020) 265:325–32. doi: 10.1016/j.jad.2020.01.045, PMID: 32090756

[176] Shirayama Y Chen AC-H Nakagawa S Russell DS Duman RS . Brain-derived neurotrophic factor produces antidepressant effects in behavioral models of depression. J Neurosci. (2002) 22:3251–61. doi: 10.1523/JNEUROSCI.22-08-03251.2002, PMID: 11943826 PMC6757539

[177] Schmidt HD Duman RS . Peripheral BDNF produces antidepressant-like effects in cellular and behavioral models. Neuropsychopharmacology. (2010) 35:2378–91. doi: 10.1038/npp.2010.114, PMID: 20686454 PMC2955759

[178] Alitalo O Kohtala S Rosenholm M Saarreharju R González-Hernández G Sarparanta M . Nitrous oxide induces hypothermia and TrkB activation: Maintenance of body temperature abolishes antidepressant-like effects in mice. Neuropharmacology. (2024) 261:110172. doi: 10.1016/j.neuropharm.2024.110172, PMID: 39362627

[179] Kohtala S Theilmann W Rosenholm M Penna L Karabulut G Uusitalo S . Cortical excitability and activation of trkB signaling during rebound slow oscillations are critical for rapid antidepressant responses. Mol Neurobiol. (2019) 56:4163–74. doi: 10.1007/s12035-018-1364-6, PMID: 30288695 PMC6505519

[180] Callaghan CK Rouine J Dean RL Knapp BI Bidlack JM Deaver DR . Antidepressant-like effects of 3-carboxamido seco-nalmefene (3CS-nalmefene), a novel opioid receptor modulator, in a rat IFN-α-induced depression model. Brain Behav Immun. (2018) 67:152–62. doi: 10.1016/j.bbi.2017.08.016, PMID: 28844812

[181] Koyama T Fukuda K . Involvement of the kappa-opioid receptor in nitrous oxide-induced analgesia in mice. J Anesth. (2010) 24:297–9. doi: 10.1007/s00540-010-0886-5, PMID: 20157832

[182] Ohgami Y Chung E Quock RM . Nitrous oxide-induced NO-dependent neuronal release of β-endorphin from the rat arcuate nucleus and periaqueductal gray. Brain Res. (2010) 1366:38–43. doi: 10.1016/j.brainres.2010.10.010, PMID: 20937263 PMC2993853

[183] Marcus E . Nitrous oxide in the treatment of depression: A brief review. Am J Psychiatry Residents’ J. (2024) 20:18–21. doi: 10.1176/appi.ajp-rj.2024.200207

[184] Goldstein BI Schaffer A Wang S Blanco C . Excessive and premature new-onset cardiovascular disease among adults with bipolar disorder in the US NESARC cohort. J Clin Psychiatry. (2015) 76:163–9. doi: 10.4088/JCP.14m09300, PMID: 25742203

[185] Celebi G Gocmez SS Ozer C Duruksu G Yazır Y Utkan T . Propolis prevents vascular endothelial dysfunction by attenuating inflammation and oxidative damage in the chronic unpredictable stress model of depression in rats. J Pharm Pharmacol. (2023) 75:1418–29. doi: 10.1093/jpp/rgad071, PMID: 37579320

[186] Ishizaki J Yamamoto H Takahashi T Takeda M Yano M Mimura M . Changes in regional cerebral blood flow following antidepressant treatment in late-life depression. Int J Geriatr Psychiatry. (2008) 23:805–11. doi: 10.1002/gps.1980, PMID: 18214999

[187] Almeida JRC Mourao-Miranda J Aizenstein HJ Versace A Kozel FA Lu H . Pattern recognition analysis of anterior cingulate cortex blood flow to classify depression polarity. Br J Psychiatry. (2013) 203:310–1. doi: 10.1192/bjp.bp.112.122838, PMID: 23969484 PMC3787302

[188] Wei W Karim HT Lin C Mizuno A Andreescu C Karp JF . Trajectories in cerebral blood flow following antidepressant treatment in late-life depression: support for the vascular depression hypothesis. J Clin Psychiatry. (2018) 79:18m12106. doi: 10.4088/JCP.18m12106, PMID: 30358242 PMC6419103

[189] Darwish D Kumar P Urs K Dave S . Inhaled anesthetics: Beyond the operating room. J Clin Med. (2024) 13:7513. doi: 10.3390/jcm13247513, PMID: 39768435 PMC11679802

[190] Guo Z Zhao F Wang Y Wang Y Geng M Zhang Y . Sevoflurane exerts an anti-depressive action by blocking the HMGB1/TLR4 pathway in unpredictable chronic mild stress rats. J Mol Neurosci. (2019) 69:546–56. doi: 10.1007/s12031-019-01380-2, PMID: 31368063

[191] Feng M Cheng S Fang Y Lv L Guo P Wang S . Augmentation of Sevoflurane inhalation for treatment-resistant depression with different features: A case series. Asian J Psychiatr. (2023) 82:103495. doi: 10.1016/j.ajp.2023.103495, PMID: 36739717

[192] Wu M Li A Guo Y Cao F You S Cao J . GABAergic neurons in the nucleus accumbens core mediate the antidepressant effects of sevoflurane. Eur J Pharmacol. (2023) 946:175627. doi: 10.1016/j.ejphar.2023.175627, PMID: 36868292

[193] McGinty VB Lardeux S Taha SA Kim JJ Nicola SM . Invigoration of reward seeking by cue and proximity encoding in the nucleus accumbens. Neuron. (2013) 78:910–22. doi: 10.1016/j.neuron.2013.04.010, PMID: 23764290 PMC3954588

[194] Francis TC Chandra R Friend DM Finkel E Dayrit G Miranda J . Nucleus accumbens medium spiny neuron subtypes mediate depression-related outcomes to social defeat stress. Biol Psychiatry. (2015) 77:212–22. doi: 10.1016/j.biopsych.2014.07.021, PMID: 25173629 PMC5534173

[195] Wang Z Dong J Zhang M Wang S Wu J Wang S . Sevoflurane-induced overexpression of extrasynaptic α5-GABAAR via the RhoA/ROCK2 pathway impairs cognitive function in aged mice. Aging Cell. (2024) 23:e14209. doi: 10.1111/acel.14209, PMID: 38825816 PMC11488297

[196] Yang J Ju L Jia M Zhang H Sun X Ji M . Subsequent maternal separation exacerbates neurobehavioral abnormalities in rats neonatally exposed to sevoflurane anesthesia. Neurosci Lett. (2017) 661:137–42. doi: 10.1016/j.neulet.2017.09.063, PMID: 28982596 PMC5808428

[197] Bao W-W Xu W Pan G-J Wang T-X Han Y Qu W-M . Nucleus accumbens neurons expressing dopamine D1 receptors modulate states of consciousness in sevoflurane anesthesia. Curr Biol. (2021) 31:1893–1902.e5. doi: 10.1016/j.cub.2021.02.011, PMID: 33705720

[198] Gao S-Q Chen J-Q Zhou H-Y Luo L Zhang B-Y Li M-T . Thrombospondin1 mimics rapidly relieve depression via Shank3 dependent uncoupling between dopamine D1 and D2 receptors. iScience. (2023) 26:106488. doi: 10.1016/j.isci.2023.106488, PMID: 37091229 PMC10119609

[199] Noori M Hasbi A Sivasubramanian M Milenkovic M George SR . Maternal separation model of postpartum depression: potential role for nucleus accumbens dopamine D1-D2 receptor heteromer. Neurochem Res. (2020) 45:2978–90. doi: 10.1007/s11064-020-03145-5, PMID: 33057844

[200] Wang S Cheng S Feng M Guo P Qian M Shen X . Sevoflurane augmentation in treatment-resistant depression: a clinical case study. Ther Adv Psychopharmacol. (2020) 10:2045125320957126. doi: 10.1177/2045125320957126, PMID: 35186257 PMC8851135

[201] Langer G Neumark J Koinig G Graf M Schönbeck G . Rapid psychotherapeutic effects of anesthesia with isoflurane (ES narcotherapy) in treatment-refractory depressed patients. Neuropsychobiology. (1985) 14:118–20. doi: 10.1159/000118216, PMID: 3831799

[202] Langer G Karazman R Neumark J Saletu B Schönbeck G Grünberger J . Isoflurane narcotherapy in depressive patients refractory to conventional antidepressant drug treatment. A double-blind comparison with electroconvulsive treatment. Neuropsychobiology. (1995) 31:182–94. doi: 10.1159/000119190, PMID: 7659199

[203] Zhang S-S Tian Y-H Jin S-J Wang W-C Zhao J-X Si X-M . Isoflurane produces antidepressant effects inducing BDNF-TrkB signaling in CUMS mice. Psychopharmacol (Berl). (2019) 236:3301–15. doi: 10.1007/s00213-019-05287-z, PMID: 31197433

[204] Jung S Zimin PI Woods CB Kayser E-B Haddad D Reczek CR . Isoflurane inhibition of endocytosis is an anesthetic mechanism of action. Curr Biol. (2022) 32:3016–3032.e3. doi: 10.1016/j.cub.2022.05.037, PMID: 35688155 PMC9329204

[205] Brown PL Zanos P Wang L Elmer GI Gould TD Shepard PD . Isoflurane but not halothane prevents and reverses helpless behavior: A role for EEG burst suppression? Int J Neuropsychopharmacol. (2018) 21:777–85. doi: 10.1093/ijnp/pyy029, PMID: 29554264 PMC6070045

[206] Antila H Ryazantseva M Popova D Sipilä P Guirado R Kohtala S . Isoflurane produces antidepressant effects and induces TrkB signaling in rodents. Sci Rep. (2017) 7:7811. doi: 10.1038/s41598-017-08166-9, PMID: 28798343 PMC5552878

[207] Short B Fong J Galvez V Shelker W Loo CK . Side-effects associated with ketamine use in depression: a systematic review. Lancet Psychiatry. (2018) 5:65–78. doi: 10.1016/S2215-0366(17)30272-9, PMID: 28757132

[208] Davidson AJ Disma N de Graaff JC Withington DE Dorris L Bell G . Neurodevelopmental outcome at two years of age after general anaesthesia and awake-regional anaesthesia in infancy (GAS): an international multicentre, randomised controlled trial. Lancet. (2016) 387:239–50. doi: 10.1016/S0140-6736(15)00608-X, PMID: 26507180 PMC5023520

[209] Sun LS Li G Miller TL Salorio C Byrne MW Bellinger DC . Association between a single general anesthesia exposure before age 36 months and neurocognitive outcomes in later childhood. JAMA. (2016) 315:2312–20. doi: 10.1001/jama.2016.6967, PMID: 27272582 PMC5316422

[210] Evered L Silbert B Knopman DS Scott DA DeKosky ST Rasmussen LS . Recommendations for the nomenclature of cognitive change associated with anaesthesia and surgery-2018. Br J Anaesth. (2018) 121:1005–12. doi: 10.1016/j.bja.2017.11.087, PMID: 30336844 PMC7069032

[211] Abdallah CG Sanacora G Duman RS Krystal JH Ketamine and rapid-acting antidepressants: a window into a new neurobiology for mood disorder therapeutics. Nat Rev Neurosci. (2018) 19:480–90. doi: 10.1038/s41583-018-0028-z, PMID: 25341010 PMC4428310

